# Repetitive Transcranial Magnetic Stimulation for Auditory Verbal Hallucinations in Schizophrenia: A Randomized Clinical Trial

**DOI:** 10.1001/jamanetworkopen.2024.44215

**Published:** 2024-11-11

**Authors:** Qiang Hua, Lu Wang, Kongliang He, Jinmei Sun, Wenqiang Xu, Li Zhang, Yanghua Tian, Kai Wang, Gong-Jun Ji

**Affiliations:** 1 Department of Neurology, The First Affiliated Hospital of Anhui Medical University, Hefei, China; 2 Department of Psychology and Sleep Medicine, The Second Affiliated Hospital of Anhui Medical University, Hefei, China; 3 School of Mental Health and Psychological Sciences, Anhui Medical University, Hefei, China; 4 Anhui Province Key Laboratory of Cognition and Neuropsychiatric Disorders, Hefei, China; 5 Collaborative Innovation Centre of Neuropsychiatric Disorder and Mental Health, Hefei, China; 6 Affiliated Psychological Hospital of Anhui Medical University, Hefei, China; 7 Institute of Artificial Intelligence, Hefei Comprehensive National Science Center, Hefei, China; 8 Anhui Institute of Translational Medicine, Hefei, China

## Abstract

**Importance:**

Auditory verbal hallucinations (AVH) are a common symptom of schizophrenia, increasing the patient’s risks of suicide and violence. Repetitive transcranial magnetic stimulation (rTMS) is a potential treatment for AVH.

**Objective:**

To investigate the effect of imaging-navigated rTMS on AVH in patients with schizophrenia.

**Design, Setting, and Participants:**

This 6-week, double-blind, sham-controlled, randomized clinical trial was performed at the Anhui Mental Health Center, Hefei, China, from September 1, 2016, to August 31, 2021. Participants included 66 patients with AVH and schizophrenia. Data were analyzed from May 1, 2022, to March 31, 2023.

**Interventions:**

Participants were randomly assigned 1:1 to either imaging-navigated active or sham rTMS over the left temporoparietal junction for 2 weeks.

**Main Outcomes and Measures:**

The primary outcome measured improvements in AVH from baseline to week 2 and week 6 using the Auditory Hallucination Rating Scale (AHRS) scores. In addition, the TMS-induced electric field strength was used to estimate improvements in AVH as a secondary outcome.

**Results:**

A total of 62 participants (33 women [53%]; mean [SD] age, 27.4 [9.2] years) completed the 2-week treatments. Of these, 32 were randomized to the active rTMS group (18 women [56%]; mean [SD] age, 26.9 [9.2] years) and 30 to the sham treatment group (15 women [50%]; mean [SD] age, 27.8 [9.4] years). In the intention-to-treat analyses, patients receiving active rTMS showed a significantly greater reduction in AHRS scores compared with those receiving sham treatment at week 2 (difference, 5.96 [95% CI, 3.42-8.50]; *t* = 4.61; *P* < .001; Cohen *d,* 1.17 [95% CI, 0.62-1.71]). These clinical effects were sustained at week 6. Additionally, a stronger TMS-induced electric field within a predefined AVH brain network was associated with greater reductions in AHRS scores (*B* = 3.12; *t* = 3.58; *P* = .002). No serious adverse event was observed.

**Conclusions and Relevance:**

The findings of this randomized clinical trial suggest that imaging-navigated rTMS may effectively and safely alleviate AVH in patients with schizophrenia. Findings also suggest that the electric field strength in the individualized AVH network is a vital parameter for optimizing the efficacy of the rTMS protocol.

**Trial Registration:**

ClinicalTrials.gov Identifier: NCT02863094

## Key Points

**Question:**

Is imaging-navigated repetitive transcranial magnetic stimulation (rTMS) an effective treatment for auditory verbal hallucinations (AVH) in patients with schizophrenia?

**Findings:**

In this randomized clinical trial of 62 patients with schizophrenia and AVH, active rTMS was superior to sham treatment in alleviating AVH symptoms, and the effects were maintained throughout the follow-up period. Furthermore, the strength of the TMS-induced electric field was independently associated with AVH symptom improvements.

**Meaning:**

The findings of this study indicate that imaging-navigated rTMS is an effective and safe treatment for AVH in patients with schizophrenia.

## Teaser

This randomized clinical trial tests the efficacy of imaging-navigated repetitive transcranial magnetic stimulation (TMS) in reducing auditory verbal hallucinations in patients with schizophrenia.

## Introduction

Auditory verbal hallucinations (AVH), generally defined as hearing nonexistent spoken voices, are a typical symptom of schizophrenia.^1^ Approximately 70% to 80% of patients with schizophrenia have AVH at initial presentation,^2,3^ and of these, 25% to 30% of AVH are nonresponsive to antipsychotic medications.^4^ The persistence of AVH can significantly diminish the quality of life^5^ and elevate the risk of suicide^6^ and violence.^7^ Consequently, effective alternative therapies are urgently needed to treat AVH in patients with schizophrenia. To this end, numerous neuroimaging studies have been tested to identify the neural correlates of AVH.^8,9^

Among the abundant brain regions or networks that may be involved in the generation of AVH in patients with schizophrenia,^10^ the left temporoparietal junction (TPJ) has attracted particular attention because of its potential for reducing AVH in clinical treatments such as repetitive transcranial magnetic stimulation (rTMS).^11,12,13,14^ Most of the rTMS studies treating AVH in patients with schizophrenia^15,16,17,18,19^ localized the left TPJ according to the international 10-20 system of electroencephalography. However, the TPJ is a heteromodal association cortex consisting of heterogeneous subregions^20,21,22,23^ with high interindividual variability.^24^ The 10-20 system is not sufficiently accurate to guide the TMS coil to a predefined TPJ subregion.^25^ As a result, the stimulation may be delivered to functionally distinct TPJ subregions on different treatment days.^26^ This imprecise stimulation may fail to accomplish an accumulated effect to alleviate AVH symptoms in patients with schizophrenia.^15^ To overcome this shortcoming, our group precisely guided the coil and monitored its online position using individual magnetic resonance imaging (MRI) of the brain in an open-label rTMS study, which significantly decreased AVH in patients with schizophrenia.^13^

Despite the success of the imaging-navigated rTMS treatment at the group level, our group also noted high outcome variability among individuals.^13^ This variability could be attributed to the heterogeneous neuroanatomy (eg, cortical folding pattern) effect on the TMS-induced electric field (e-field). The imaging-navigated procedure ensured the spatial alignment between coil and target, but the e-field shape and strength were differentially distorted by the neuroanatomy of different individuals.^27,28,29^ E-field variability in local areas and brain networks has been used to explain the treatment effects. For example, the e-field on the stimulation site^30^ or within the depression-related network^31^ was associated with improvements in depressive symptoms. These local and network explanations are not mutually exclusive, and both may be related to the AVH outcome variability in patients with schizophrenia.

In this study, we designed a randomized, double-blind, sham-controlled clinical trial to test the efficacy of imaging-navigated rTMS in reducing AVH in patients with schizophrenia. To estimate the outcome variability, we correlated individual AVH improvements with the simulated e-field strength in the stimulation site and a predefined AVH network. We defined the AVH network as a circuit composed of brain regions that were associated with AVH.^32,33^

## Methods

This single-site, randomized, double-blind, sham-controlled clinical trial was performed at the Anhui Mental Health Center, Hefei, China, from September 1, 2016, to August 31, 2021. The Institutional Ethics Committee of Anhui Medical University approved the protocol, which is found in Supplement 1. This study followed the Consolidated Standards of Reporting Trials (CONSORT) reporting guideline. All participants provided written, informed consent to participate.

### Participants

Patients with schizophrenia were recruited through posted flyers and physician referrals at Anhui Mental Health Center. The inclusion criteria for patients consisted of the following: (1) diagnosis by independent psychiatrists using the Structured Clinical Interview for the *Diagnostic and Statistical Manual of Mental Disorders* (Fourth Edition); (2) age of 18 to 60 years; (3) medication-resistant AVH (defined as an insufficient response to antipsychotic agents for AVH, administered at adequate dosages for at least 6 weeks^34,35^); and (4) a stable dosage of antipsychotic medication for at least 2 weeks before inclusion and retention of this stable dose for the duration of the study. Exclusion criteria consisted of the following: (1) other comorbid mental illnesses or histories; (2) pregnancy; (3) a history of severe head trauma or neurological disease; (4) focal brain lesions on T1- or T2-weighted fluid-attenuated inversion-recovery MRI; (5) a history of rTMS or electroconvulsive therapy; (6) current severe suicidal thoughts or behavior; and (7) metal objects in the head or any other contraindication to MRI.

### Randomization and Blinding

Participants were randomly assigned to an active treatment or a sham treatment group at a 1:1 ratio using a random number generator by an unblinded investigator (G.-J. J.) who was not involved with the study ratings or analysis. Patients, clinical raters, and all personnel responsible for the clinical care of patients were blinded to the treatment assignments. To measure blinding integrity, the participants were asked at week 6 to guess the group to which they were randomly assigned and were subsequently informed of their respective treatment conditions.

### MRI Data Acquisition

Structural and resting-state functional MRI (rs-fMRI) data were acquired for each patient at baseline (before the first session of rTMS treatment) using a 3.0-T scanner (Discovery MR750; GE Healthcare) at the University of Science and Technology of China in Hefei. The specific MRI parameters and scanning requirements are presented in eMethods 1 in Supplement 2.

### Imaging-Navigated Treatment

Treatment was performed using a transcranial magnetic stimulator (Rapid^2^; Magstim) with a 70-mm air-cooled figure-of-8 coil. Participants received 3 daily sessions of rTMS treatment for 2 weeks (14 consecutive days). The specific rTMS protocol followed that of the previous open-label study from our group^13^ (eMethods 2 in Supplement 2).

Sham treatments were delivered with the same rTMS protocol using a sham coil (Magstim) that was identical in appearance to the real one. The only difference was that the sham coil generated only sound and sensations on the scalp similar to those of the real coil but no current, which prevented patients from identifying the rTMS group.

### Electric Field Modeling

We calculated realistic estimates of the e-fields induced by the TMS coil in our intervention based on the finite elements method using the SimNIBS software package, version 4.0.0 (Danish Research Center for Magnetic Resonance and the Technical University of Denmark).^36^ We computed the head mesh for each patient using their individual T1- and T2-weighted images.^37^ Brain images were segmented into 5 tissue types (white matter, gray matter, cerebrospinal fluid, bone, and skin). We then estimated the e-field strength using default tissue conductivities and coil-to-scalp distances.^38^ The position and orientation of the TMS coil for each patient were exported from the neuronavigation system. The stimulation intensity (the derivative of the current flowing across the inductor [di/dt] = 165 A/μs) was provided by the transcranial magnetic stimulator manufacturer.

We calculated e-field strength for 2 regions of interest, target and AVH network regions. The e-field strength of the TMS target region was the mean strength within the sphere target of TPJ. The e-field strength in each patient’s AVH network was computed in 3 steps (eFigure 1 in Supplement 2). First, we computed the group-level AVH network defined as the connectivity map of a sphere seed in the cerebellum (Montreal Neurological Institute coordinates, 1, −50, −28). This seed in the cerebellum was the hub region of the AVH network identified by a prior lesion network mapping study.^32^ Specifically, we computed cerebellum seed–to–whole brain functional connectivity on the rs-fMRI data of 652 healthy individuals as part of a previous protocol (see the demographic information and MRI parameters in eMethods 3 in Supplement 2), then performed a 1-sample *t* test on 652 connectivity maps to obtain the group-level network. Second, we individualized this group-level AVH network by using each patient’s rs-fMRI data acquired at baseline. We used the positive part of the group-level AVH network as a seed map and computed its functional connectivity (Pearson correlation) with each voxel in the patient’s brain. Voxels positively correlated with the seed map (uncorrected *P* < .05) constituted the individualized AVH network of each patient. Finally, we calculated the mean effective e-field strength (>10 V/m)^39^ within individualized AVH networks.

### Clinical Symptom Assessments

All assessments were performed by a trained psychiatrist (L.W.) blinded to the patients’ rTMS conditions. Clinical symptom assessments were performed at baseline, week 2 (after treatment), and week 6 (follow-up). Any adverse events related to rTMS (eg, significant discomfort, pain, or harm caused by the intervention) were recorded throughout the study from the first stimulation session to the last follow-up visit at week 6.

The primary outcome measure was the change in the Auditory Hallucination Rating Scale (AHRS) scores from baseline to weeks 2 and 6. Secondary outcomes included the Positive and Negative Syndrome Scale (PANSS), Hamilton Anxiety Rating Scale with 14 items, Hamilton Depression Rating Scale with 17 items, and response rates at weeks 2 and 6. Response to rTMS treatment was defined as a reduction of 25% or more from baseline in the AHRS score.^13^ We estimated the TMS-induced e-field strength using baseline structural brain images and tested whether the e-field strength could estimate the improvement of AVH symptoms after treatment.

### Statistical Analysis

Data were analyzed from May 1, 2022, to March 31, 2023. The sample size was estimated for a power of 80% and a 2-tailed α of .05 for assessing AVH improvements. We calculated the sample size according to the effect size of 0.76 in low-frequency rTMS in the treatment of resistant AVH, the effect size reported in a previous meta-analysis.^40^ In addition, considering a 10% attrition rate, each group was required to include 33 participants.

All patients who underwent randomization and received at least 1 active or sham treatment were classified as the intention-to-treat (ITT) analysis set. Notably, 4 participants discontinued trials before baseline assessments and first treatments, so their data were unavailable for analysis. Consequently, the main analyses were conducted on the ITT analysis set, excluding these 4 participants.

We used SPSS statistical software for Windows, version 23.0 (IBM Corporation) for statistical analysis. Baseline demographic and clinical characteristics were compared between the 2 groups using χ^2^ tests for categorical variables. Continuous variables were compared using independent-sample *t* tests and Mann-Whitney tests, depending on the normality of the data. The normality of continuous variables was tested using the Shapiro-Wilks test, and if normality was violated, we used a Mann-Whitney test. For all continuous outcome measures, we used linear mixed-effects models to test the treatment efficacies. Time, group, and time-by-group interaction were included in the model as fixed effects. The individual participant intercept was included in the model as a random effect. For categorical outcome measures, we used χ^2^ tests (or Fisher exact tests if the expected frequencies in any cell of the contingency table were less than 1) to compare the differences between groups. Two-sided *P* < .05 was considered statistically significant. Effect sizes were calculated as Cohen *d* and odds ratios (ORs) with 95% CIs for continuous and binary outcomes, respectively. We also provided the number needed to treat (NNT) to assess treatment effectiveness.^41^

To assess whether the TMS-induced e-field strength was associated with clinical efficacy, hierarchical multiple regression analysis was conducted.^42^ Hierarchical multiple regression analysis included at least 2 models. Model 1 represented a control model with confounders. Model 2 included both confounders and estimation factors. In this study, model 1 included the reduction in AHRS scores as the dependent variable and baseline data as confounders (ie, sex, age, education years, illness duration, olanzapine equivalent, and baseline AHRS scores). In model 2, the estimation factors were TMS-induced e-field strength within the TPJ target region or AVH network. The estimation abilities of the 2 models were compared using an *F* test. We then used a 1-sample *t* test to assess the contribution of the factors in estimating the dependent variable. Notably, the sham coil in the sham treatment group did not generate an e-field in the cortex. Thus, it was not appropriate to include both active and sham treatment data in 1 regression model, although we could simulate the e-field for the sham treatment group.

## Results

### Participants

**Figure 1.**
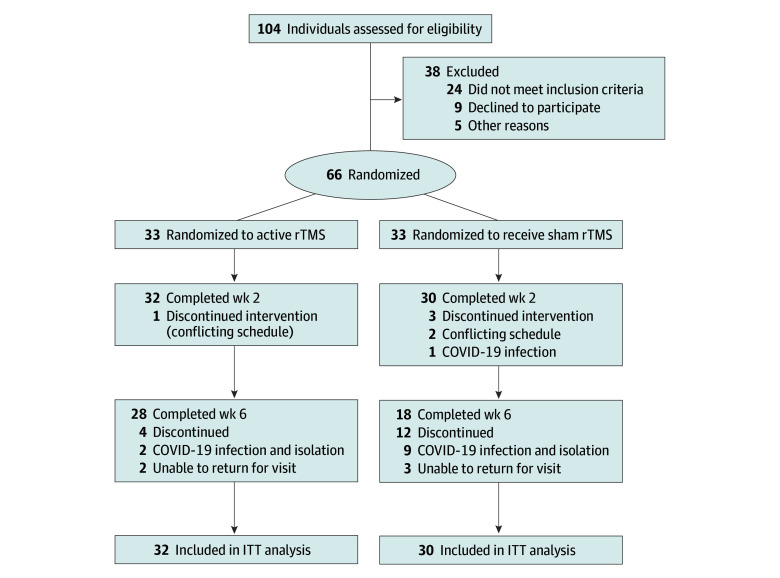
Patient Flow Diagram A total of 62 patients were included in the intention-to-treat (ITT) analysis. rTMS indicates repetitive transcranial magnetic stimulation.

Of 104 participants, 38 were excluded for several reasons and 66 (31 men [47%] and 35 women [53%]; mean [SD] age, 27.4 [9.0] years) were randomized to the active group (n = 33) or sham treatment group (n = 33). A total of 62 participants (29 men [47%] and 33 women [53%]; mean [SD] age, 27.4 [9.2] years) completed the 2-week treatments. Of these, 32 participants were randomized to the active rTMS group (14 men [44%] and 18 women [56%]; mean [SD] age, 26.9 [9.2] years) and 30 to the sham treatment group (15 men [50%] and 15 women [50%]; mean [SD] age, 27.8 [9.4] years). There was no significant difference in baseline demographic and clinical variables between the active rTMS and sham treatment groups (Table 1). There was a significant difference in the dropout rates between the active rTMS and sham treatment groups during the follow-up phase (4 in the active rTMS group and 12 in the sham treatment group; χ^2^ = 6.12; *P* = .02). Participant flow is illustrated in Figure 1.

**Table 1.** Patient Demographic and Clinical Characteristics

Characteristic	Patient group, mean (SD)		Statistics
	Sham treatment (n = 30)	Active rTMS (n = 32)	
**Demographic characteristics**			
Age, mean (SD), y	27.8 (9.4)	26.9 (9.2)	Mann-Whitney test = −0.67
Sex, No. (%)			
Men	15 (50)	14 (44)	
Women	15 (50)	18 (56)	χ^2^ = 0.24
Educational level, y	11.93 (2.63)	11.38 (3.39)	*t* = −0.72^a^
Illness duration, y	6.98 (6.23)	7.09 (4.62)	Mann-Whitney test = −0.64
Olanzapine equivalent, mg	21.82 (20.33)	26.73 (15.71)	Mann-Whitney test = −1.68
**Baseline clinical characteristics**			
AHRS^b^	24.47 (6.62)	26.25 (6.44)	*t* = 1.08^a^
PANSS total^c^	64.00 (15.15)	68.75 (17.18)	*t* = 1.15^a^
PANSS positive^d^	17.20 (5.71)	18.81 (5.92)	*t* = 1.09^a^
PANSS negative^d^	15.17 (4.84)	17.44 (5.38)	*t* = 1.74^a^
PANSS general^e^	31.63 (7.38)	32.50 (8.20)	*t* = 0.44^a^
Emotion			
HAMA^f^	6.20 (3.53)	6.13 (4.47)	Mann-Whitney test = −0.50
HAMD^g^	5.53 (3.66)	5.16 (2.69)	Mann-Whitney test = −0.04

Abbreviations: AHRS, Auditory Hallucination Rating Scale; HAMA, Hamilton Anxiety Rating Scale; HAMD, Hamilton Depression Rating Scale; PANSS, Positive and Negative Syndrome Scale; rTMS, repetitive transcranial magnetic stimulation.

^a^ Calculated as 2-sample *t* test.

^b^ Score range, 0 to 41; higher scores indicate more severe symptoms.

^c^ Score range, 30 to 210; higher scores indicate more severe symptoms.

^d^ Score range, 7 to 49; higher scores indicate more severe symptoms.

^e^ Score range, 16 to 112; higher scores indicate more severe symptoms.

^f^ Score range, 0 to 56; higher scores indicate more severe symptoms.

^g^ Score range, 0 to 50; higher scores indicate more severe symptoms.

### Primary Outcome

**Figure 2.**
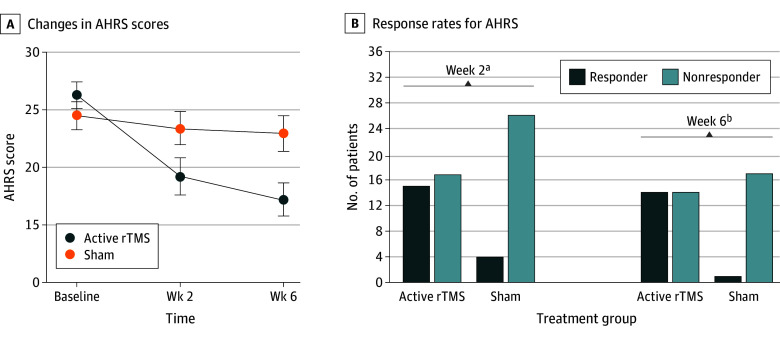
Clinical Outcomes of the Repetitive Transcranial Magnetic Stimulation (rTMS) Treatment The error bars indicate 1 standard error of the mean. AHRS indicates Auditory Hallucination Rating Scale. ^a^*P* = .004 between treatment groups. ^b^*P* = .002 between treatment groups.

The primary outcome consisted of changes in AHRS scores at weeks 2 and 6. In the ITT analysis set, linear mixed-effects model analysis revealed significant time × group interactions for the AHRS at week 2 (*F*_1,60_ = 21.20; *P* < .001) and week 6 (F_1,48.62_ = 24.52; *P* < .001). A post hoc test revealed that patients receiving active rTMS showed a greater reduction in the AHRS scores than the sham treatment group at week 2 (between-group difference, 5.96 [95% CI, 3.42-8.50]; *P* < .001; *t *= 4.61; Cohen *d*, 1.17 [95% CI, 0.63-1.71]) and at week 6 (group difference, 7.89 [95% CI, 4.77-11.01]; *P* < .001; Cohen *d*, 1.49 [95% CI, 0.82-2.16]) (Figure 2A and Table 2).

**Table 2.** Primary and Secondary Outcomes

Outcome	Patient group, mean (SD)		Sham treatment vs active rTMS group			
	Sham treatment (n = 30)	Active rTMS (n = 32)	Difference (95% CI)^a^	Time × group^b^	*P* value	Effect size, Cohen *d* (95% CI)
**Primary outcome at week 2**						
Change in AHRS^c^	−1.07 (5.09)	−7.03 (5.09)	5.96 (3.42 to 8.50)	*F*_1,60_ = 21.20	<.001	1.17 (0.63 to 1.71)
**Secondary outcomes at week 2** ^a^						
Change in PANSS total^d^	−2.17 (9.69)	−13.03 (9.67)	10.86 (6.04 to 15.68)	*F*_1,60_ = 19.51	<.001	1.12 (0.59 to 1.66)
Change in PANSS positive^e^	−1.00 (3.23)	−3.75 (3.22)	2.75 (1.14 to 4.36)	*F*_1,60_ = 11.30	.001	0.85 (0.33 to 1.37)
Change in PANSS negative^e^	−0.40 (3.51)	−3.47 (3.51)	3.07 (1.32 to 4.82)	*F*_1,60_ = 12.00	<.001	0.88 (0.35 to 1.40)
Change in PANSS general^f^	−0.77 (5.26)	−5.81 (5.26)	5.04 (2.42 to 7.66)	*F*_1,60_ = 14.13	<.001	0.96 (0.43 to 1.48)
Change in HAMA^g^	0.07 (4.00)	−2.22 (4.02)	2.29 (0.29 to 4.29)	*F*_1,60_ = 5.09	.03	0.57 (0.06 to 1.08)
Change in HAMD^h^	−0.03 (2.79)	−1.75 (2.77)	1.72 (0.33 to 3.11)	*F*_1,60_ = 5.97	.02	0.62 (0.11 to 1.13)
≥25% Improvement^i^	4 (13)	15 (47)	2.98 (1.86 to 12.04)	χ^2^ = 8.20	.004	5.74 (1.63 to 20.24)
**Primary outcome at week 6**						
Change in AHRS^c^	−0.86 (5.22)	−8.75 (5.34)	7.89 (4.77 to 11.01)	*F*_1,48.62_ = 24.52	<.001	1.49 (0.82 to 2.16)
**Secondary outcomes at week 6** ^a^						
Change in PANSS total^d^	0.73 (10.14)	−12.62 (10.27)	13.35 (7.32 to 19.38)	*F*_1,47.13_ = 18.80	<.001	1.31 (0.66 to 1.96)
Change in PANSS positive^e^	0.18 (4.16)	−4.11 (4.23)	4.29 (1.81 to 6.77)	*F*_1,48.53_ = 11.63	.001	1.02 (0.39 to 1.65)
Change in PANSS negative^e^	0.08 (3.65)	−4.05 (3.70)	4.13 (1.96 to 6.30)	*F*_1,46.47_ = 13.98	<.001	1.12 (0.49 to 1.76)
Change in PANSS general^f^	0.48 (5.35)	−4.57 (5.45)	5.05 (1.86 to 8.24)	*F*_1,47.74_ = 9.54	.003	0.93 (0.31 to 1.56)
Change in HAMA^g^	−0.06 (4.29)	−3.14 (4.50)	2.72 (−0.04 to 5.48)	*F*_1,46.24_ = 5.21	.03	0.70 (0.07 to 1.32)
Change in HAMD^h^	0.43 (2.72)	−1.88 (2.80)	2.31 (0.64 to 3.98)	*F*_1,42.15_ = 7.39	.009	0.84 (0.20 to 1.47)
≥25% Improvement^i^	1 (6)	14 (50)	2.25 (1.56 to 7.13)	χ^2^ = 9.85	.002	17.00 (1.98 to 145.73)

Abbreviations: AHRS, Auditory Hallucination Rating Scale; HAMA, Hamilton Anxiety Rating Scale; HAMD, Hamilton Depression Rating Scale; OR, odds ratio; PANSS, Positive and Negative Syndrome Scale; rTMS, repetitive transcranial magnetic stimulation.

^a^ Changes in scale score were the estimated marginal mean from linear mixed-effects models. The differences were calculated based on the estimated marginal mean.

^b^ Time-by-group interaction of linear mixed-effects model.

^c^ Score range, 0 to 41; higher scores indicate more severe symptoms.

^d^ Score range, 30 to 210; higher scores indicate more severe symptoms.

^e^ Score range, 7 to 49; higher scores indicate more severe symptoms.

^f^ Score range, 16 to 112; higher scores indicate more severe symptoms.

^g^ Score range, 0 to 56; higher scores indicate more severe symptoms.

^h^ Score range, 0 to 50; higher scores indicate more severe symptoms.

^i^ Shows No. (%) for patient group, number needed to treat rather than difference, and OR (95% CI) rather than Cohen *d*.

### Secondary Outcomes

Participants in the active rTMS group had higher response rates than the sham treatment group at week 2 (15 of 32 [47%] vs 4 of 30 [13%]; χ^2^ = 8.20; *P* = .004; OR, 5.74 [95% CI, 1.63-20.24]; NNT, 2.98 [95% CI, 1.86-12.04]) (Table 2 and Figure 2B). The same pattern was observed at week 6 (14 of 28 [50%] vs 1 of 18 [6%]; χ^2^ = 9.85; *P* = .002; OR, 17.00 [95% CI, 1.98-145.73]; NNT, 2.25 [95% CI, 1.56-7.13]) (Table 2 and Figure 2B).

At weeks 2 and 6, we observed significant time × group interactions for the PANSS total score (*F*_1,60_ = 19.51 and *F*_1,47.13_ = 18.80, respectively [*P* < .001]) (eFigure 2 in Supplement 2), PANSS positive score (*F*_1,60_ = 11.30 and *F*_1,48.53_ = 11.63, respectively [*P* = .001]), PANSS negative score (*F*_1,60_ = 12.00 and *F*_1,46.47_ = 13.98, respectively [*P* < .001]), PANSS general score (*F*_1,60_ = 14.13 [*P* < .001] and *F*_1,47.74_ = 9.54 [*P* = .003], respectively), Hamilton Anxiety Rating Scale (*F*_1,60_ = 5.09 and *F*_1,46.24_ = 5.21, respectively [*P* = .03]), and Hamilton Depression Rating Scale (*F*_1,60_ = 5.97 [*P* = .02] and *F*_1,42.15_ = 7.39 [*P* = .009], respectively) (Table 2). Post hoc tests revealed that patients receiving active rTMS showed greater reductions in these scale scores than the sham treatment group at week 2 and week 6 (Table 2). We additionally defined the response to rTMS treatment as a reduction of 25% or more from baseline in the PANSS total score (for detailed results, see eResults 1 and eFigure 2 in Supplement 2). The missing data analysis and sensitivity analysis were detailed in eMethods 4, eResults 2, and eTable 1 in Supplement 2.

### Estimating Clinical Efficacy

**Figure 3.**
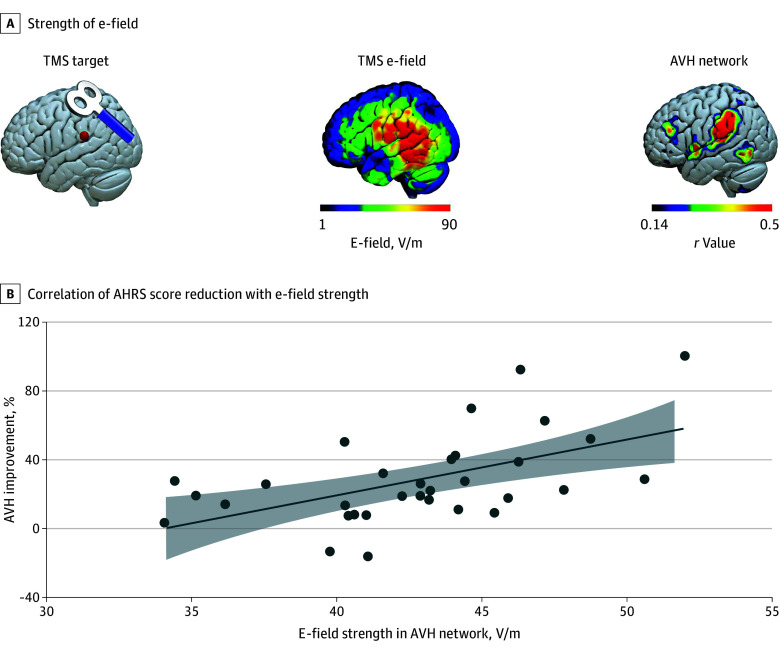
Estimating Clinical Efficacy by Electric Field (E-Field) Strength A, Illustration of stimulation target on the left temporoparietal junction (TPJ) (left), distribution of the transcranial magnetic stimulation (TMS)–induced e-field in 1 patient (middle), and the individualized auditory verbal hallucination (AVH) network (right). B, The Auditory Hallucination Rating Scale (AHRS) reductions were positively correlated with e-field strength within each patient’s individualized AVH network (*r* = 0.54; *P* = .001) but not with the e-field strength within the TPJ target (*r* = 0.14; *P* = .44). Solid line indicates the regression line; shading, 95% CI for the regression line.

Model 1 did not exhibit a significant ability to estimate outcome (*R*^2^ = 0.34; *F* = 2.10; *P* = .09). The reduction in AHRS scores was positively correlated with e-field strength within the AVH network (*r* = 0.54; *P* = .001) (Figure 3) but not with e-field strength within the target region (*r* = 0.14; *P* = .44). Therefore, the e-field strength within the AVH network was added into model 2 as a variable of estimation, while the e-field strength within the target region was not included. Model 2 exhibited a significant ability to estimate outcomes (*R*^2^ = 0.57; *F* = 4.48; *P* = .003). The e-field strength within the AVH network significantly enhanced the ability of the model to estimate outcomes (change in *R*^2^ = 0.23; *F* = 12.80; *P* = .002; *B* = 3.12; *t* = 3.58; *P* = .002). Detailed regression coefficients of hierarchical multiple regression are given in eTable 2 in Supplement 2. Additionally, analysis of the ability of the e-field strength to estimate outcomes in the sham treatment group is detailed in the eResults 3 and eFigure 3 in Supplement 2. To test the robustness of the estimation findings, we reconducted the hierarchical multiple regression analysis using different confounders (details in eResults 4 and eTables 3 and 4 in Supplement 2).

### Adverse Effects and Safety

There was no significant difference in the rates of adverse events between the active rTMS and sham treatment groups throughout the study (eTable 5 in Supplement 2). The most common adverse event was sleepiness. All adverse effects were tolerable and gradually disappeared on cessation of treatment. No serious adverse events were reported.

### Integrity of Blinding

Eighteen of 32 participants in the active rTMS group and 11 of 30 in the sham treatment group correctly guessed their actual group. There was no statistically significant difference between groups (χ^2^ = 2.39; *P* = .12).

## Discussion

In this randomized clinical trial, we determined the effects of imaging-navigated rTMS for treating AVH in patients with schizophrenia. The treatment protocol was tolerable to patients, and no serious adverse events were reported. The primary outcome, hallucination symptoms, showed a significantly higher decrease in the active treatment group than in the sham treatment group after 2 weeks of treatment, which persisted to week 6. The e-field strength on the AVH network was independently associated with AVH outcomes and was specific to active stimulations compared with sham stimulations.

Although meta-analysis has suggested that rTMS is a promising treatment for AVH in patients with schizophrenia,^11,43^ there has been considerable variation in the stimulation parameters across different studies. We did not know how to establish an effective parameter. Clinical guidelines also defined the efficacy at a “possible level.”^44^ In a pilot open-label study,^13^ our group showed the possible efficiency of an MRI-navigated protocol for treating AVH symptoms. This efficiency was further validated by the current sham-control clinical trial. Similarly to the pilot study (response ratio of 37.5%),^13^ we found that 47% (15 of 32) of patients were responsive to active stimulations. This high response ratio may be attributed to precise guiding and online monitoring of the coil position on the left TPJ. This is consistent with the positive findings in fMRI-guided studies when stimulating areas around the left TPJ.^14,45^ However, in studies where the target was not restricted around the TPJ, despite precise localizations, no significant efficacy was found compared with a placebo effect.^34,46^ This is consistent with the recently identified schizophrenia network synthesized from 143 structural and functional MRI studies.^47^ This network highlighted 2 superficial cortical areas within the stimulation depth of rTMS. One was the TPJ. The other was the dorsal prefrontal cortex (DLPFC), which was confirmed by another clinical trial.^48^ Future studies may adopt the precise locations of the TPJ and DLPFC in these studies^13,48^ and perform dual-site trials to enhance clinical efficacy.

The beneficial effects of treatment were not limited to AVH. We also found significant active rTMS effects on PANSS total and subscale scores. This is consistent with the findings of the previous open-label study.^13^ This improvement can be attributed to 2 factors. First, AVH is one of the symptoms evaluated in the PANSS. For some patients, AVH is even the dominant symptom. Thus, the PANSS scores naturally decreased with the alleviation of AVH. This phenomenon has also been found in a transcranial direct-current stimulation study.^49^ Second, this stimulation protocol designed for AVH also modulated the neural correlates of other schizophrenia symptoms because the left TPJ is a hub region of AVH and also a part of the schizophrenia network.^47^ The high connectivity between the TPJ and prefrontal cortex may also mediate the modulation effect to the DLPFC hub regions of negative symptoms.^50,51^ However, the reduction in PANSS scores in the active group was below the minimum clinically meaningful difference.^52^

In contrast to the significant findings at the group level, we noticed high outcome variability between individuals in the active group. This variability was probably related to the precise yet impersonalized stimulation setting with a one-fits-all target. We selected the TPJ as the stimulation target based on previous group-level findings^13,47^ but did not consider the function variability of the TPJ among individuals.^24,53^ The same neuroanatomical site (eg, TPJ) of different persons may be involved in different functional networks.^54^ A unified TPJ site cannot represent AVH-related regions in all patients. Another parameter that needs to be controlled for each patient is the e-field pattern.^55^ TMS manipulates neural excitability by changing the local e-field. In the brain, the e-field is reshaped by individual tissue distributions, such as the coil-to-cortex distances and cortical gyrifications. As a result, TMS may lose its focality and modulate cortices outside the predefined target.^28^ In hierarchical multiple regression analysis, we considered these 2 factors individually to identify factors associated with outcome variability. We found that the individual e-field strength of the individualized AVH network, rather than the TPJ target, was associated with AVH improvement. This is consistent with other transcranial electric stimulation studies, which reported that e-field distributions within different brain networks were associated with particular symptoms and cognitive outcomes.^31,56^

### Strengths and Limitations

The strengths of our study include using image navigation to keep stimulations at the same site on different treatment days and identifying the interaction of the e-field and AVH network as a factor associated with symptom improvements. There are also some limitations to consider. First, this trial was conducted at a single center with a small sample size. A larger multisite trial is required to replicate our findings and determine the effects of more clinical factors on treatment efficacy. Second, the rTMS effect on cognitive function was not tested. We did not include cognition measures in this study because the previous open-label study showed that the same stimulation protocol had no significant effect on cognitive function.^13^ Third, although we found that the therapeutic effects of the active group were still evident at the end of the study, a longer follow-up period is required to determine how long the treatment effects can be sustained. This information is vital for designing a continuous treatment schedule. Fourth, the resting motor threshold of each participant was measured only at baseline. Considering that the resting motor threshold varied significantly across days among participants receiving rTMS,^57^ future studies should test the threshold frequently to adjust the intensity of rTMS to enhance treatment efficacy. Fifth, clinical raters were blinded to the treatment assignments, but we did not assess the blinding integrity. This absence may increase the risk of detection bias. Sixth, the dropout rate in the sham treatment group was higher than in the active group at follow-up. This discrepancy should be noted in interpreting the follow-up results, since the small sample size in the sham treatment group may reduce the statistical power.

## Conclusions

In this randomized clinical trial, imaging-navigated rTMS on the TPJ significantly alleviated the AVH in patients with schizophrenia compared with sham treatment. The rTMS protocol was also effective for positive and negative symptoms. Future studies should consider the role of individual e-fields in individualized symptom networks to improve treatment efficacy.

## Notes

Supplement 1.
Trial Protocol

Supplement 2. **eMethods 1.** MRI Data Acquisition **eMethods 2.** Treatment Protocol **eMethods 3.** Electric Field Modeling Method **eMethods 4.** Missing Data and Sensitivity Analysis **eResults 1.** Secondary Outcomes of rTMS Treatment **eResults 2.** Sensitivity Analysis of rTMS Treatment Outcomes **eResults 3.** Estimating Clinical Efficacy in Sham Group **eResults 4.** Estimating Clinical Efficacy Using Different Confounders **eTable 1.** Outcomes of Sensitivity Analysis **eTable 2.** Regression Coefficients of Hierarchical Multiple Regression (6 Confounders) **eTable 3.** Correlations Between AHRS Reductions and Variables **eTable 4.** Regression Coefficients of Hierarchical Multiple Regression (1 Confounder) **eTable 5.** Adverse Events **eFigure 1.** Individualized AVH Network Mapping Method **eFigure 2.** Clinical Secondary Outcomes of rTMS Treatment **eFigure 3.** Estimating Clinical Efficacy in Sham Group
eReferences

Supplement 3.
Data Sharing Statement

## References

[1] Sommer IE, Slotema CW, Daskalakis ZJ, Derks EM, Blom JD, van der Gaag M. The treatment of hallucinations in schizophrenia spectrum disorders. Schizophr Bull. 2012;38(4):704-714. doi:10.1093/schbul/sbs034 22368234 PMC3577047

[2] Andreasen NC, Flaum M. Schizophrenia: the characteristic symptoms. Schizophr Bull. 1991;17(1):27-49. doi:10.1093/schbul/17.1.27 2047788

[3] Goghari VM, Harrow M, Grossman LS, Rosen C. A 20-year multi-follow-up of hallucinations in schizophrenia, other psychotic, and mood disorders. Psychol Med. 2013;43(6):1151-1160. doi:10.1017/S0033291712002206 23034091

[4] Shergill SS, Murray RM, McGuire PK. Auditory hallucinations: a review of psychological treatments. Schizophr Res. 1998;32(3):137-150. doi:10.1016/S0920-9964(98)00052-8 9720119

[5] Slotema CW, Daalman K, Blom JD, Diederen KM, Hoek HW, Sommer IE. Auditory verbal hallucinations in patients with borderline personality disorder are similar to those in schizophrenia. Psychol Med. 2012;42(9):1873-1878. doi:10.1017/S0033291712000165 22336487

[6] Fujita J, Takahashi Y, Nishida A, . Auditory verbal hallucinations increase the risk for suicide attempts in adolescents with suicidal ideation. Schizophr Res. 2015;168(1-2):209-212. doi:10.1016/j.schres.2015.07.028 26232867

[7] Volavka J, Laska E, Baker S, Meisner M, Czobor P, Krivelevich I. History of violent behaviour and schizophrenia in different cultures: analyses based on the WHO study on Determinants of Outcome of Severe Mental Disorders. Br J Psychiatry. 1997;171:9-14. doi:10.1192/bjp.171.1.9 9328487

[8] Zmigrod L, Garrison JR, Carr J, Simons JS. The neural mechanisms of hallucinations: a quantitative meta-analysis of neuroimaging studies. Neurosci Biobehav Rev. 2016;69:113-123. doi:10.1016/j.neubiorev.2016.05.037 27473935

[9] Jardri R, Pouchet A, Pins D, Thomas P. Cortical activations during auditory verbal hallucinations in schizophrenia: a coordinate-based meta-analysis. Am J Psychiatry. 2011;168(1):73-81. doi:10.1176/appi.ajp.2010.09101522 20952459

[10] Alderson-Day B, Diederen K, Fernyhough C, . Auditory hallucinations and the brain’s resting-state networks: findings and methodological observations. Schizophr Bull. 2016;42(5):1110-1123. doi:10.1093/schbul/sbw078 27280452 PMC4988751

[11] Slotema CW, Blom JD, van Lutterveld R, Hoek HW, Sommer IE. Review of the efficacy of transcranial magnetic stimulation for auditory verbal hallucinations. Biol Psychiatry. 2014;76(2):101-110. doi:10.1016/j.biopsych.2013.09.038 24315551

[12] Hoffman RE, Wu K, Pittman B, . Transcranial magnetic stimulation of Wernicke’s and Right homologous sites to curtail “voices”: a randomized trial. Biol Psychiatry. 2013;73(10):1008-1014. doi:10.1016/j.biopsych.2013.01.016 23485015 PMC3641174

[13] Chen X, Ji GJ, Zhu C, . Neural correlates of auditory verbal hallucinations in schizophrenia and the therapeutic response to theta-burst transcranial magnetic stimulation. Schizophr Bull. 2019;45(2):474-483. doi:10.1093/schbul/sby054 29733409 PMC6403092

[14] Dollfus S, Jaafari N, Guillin O, . High-frequency neuronavigated rTMS in auditory verbal hallucinations: a pilot double-blind controlled study in patients with schizophrenia. Schizophr Bull. 2018;44(3):505-514. doi:10.1093/schbul/sbx127 29897597 PMC5890503

[15] Koops S, van Dellen E, Schutte MJ, Nieuwdorp W, Neggers SF, Sommer IE. Theta burst transcranial magnetic stimulation for auditory verbal hallucinations: negative findings from a double-blind-randomized trial. Schizophr Bull. 2016;42(1):250-257. doi:10.1093/schbul/sbv100 26221051 PMC4681555

[16] Kimura H, Kanahara N, Takase M, Yoshida T, Watanabe H, Iyo M. A randomized, sham-controlled study of high frequency rTMS for auditory hallucination in schizophrenia. Psychiatry Res. 2016;241:190-194. doi:10.1016/j.psychres.2016.04.119 27179693

[17] Paillère-Martinot ML, Galinowski A, Plaze M, . Active and placebo transcranial magnetic stimulation effects on external and internal auditory hallucinations of schizophrenia. Acta Psychiatr Scand. 2017;135(3):228-238. doi:10.1111/acps.12680 27987221

[18] Bais L, Liemburg E, Vercammen A, Bruggeman R, Knegtering H, Aleman A. Effects of low frequency rTMS treatment on brain networks for inner speech in patients with schizophrenia and auditory verbal hallucinations. Prog Neuropsychopharmacol Biol Psychiatry. 2017;78:105-113. doi:10.1016/j.pnpbp.2017.04.017 28442422

[19] Plewnia C, Zwissler B, Wasserka B, Fallgatter AJ, Klingberg S. Treatment of auditory hallucinations with bilateral theta burst stimulation: a randomized controlled pilot trial. Brain Stimul. 2014;7(2):340-341. doi:10.1016/j.brs.2014.01.001 24495662

[20] Wang J, Fan L, Zhang Y, . Tractography-based parcellation of the human left inferior parietal lobule. Neuroimage. 2012;63(2):641-652. doi:10.1016/j.neuroimage.2012.07.045 22846658

[21] Carter RM, Huettel SA. A nexus model of the temporal-parietal junction. Trends Cogn Sci. 2013;17(7):328-336. doi:10.1016/j.tics.2013.05.007 23790322 PMC3750983

[22] Schurz M, Tholen MG, Perner J, Mars RB, Sallet J. Specifying the brain anatomy underlying temporo-parietal junction activations for theory of mind: a review using probabilistic atlases from different imaging modalities. Hum Brain Mapp. 2017;38(9):4788-4805. doi:10.1002/hbm.23675 28608647 PMC6867045

[23] Donaldson PH, Rinehart NJ, Enticott PG. Noninvasive stimulation of the temporoparietal junction: a systematic review. Neurosci Biobehav Rev. 2015;55:547-572. doi:10.1016/j.neubiorev.2015.05.017 26073069

[24] Mueller S, Wang D, Fox MD, . Individual variability in functional connectivity architecture of the human brain. Neuron. 2013;77(3):586-595. doi:10.1016/j.neuron.2012.12.028 23395382 PMC3746075

[25] Sparing R, Buelte D, Meister IG, Paus T, Fink GR. Transcranial magnetic stimulation and the challenge of coil placement: a comparison of conventional and stereotaxic neuronavigational strategies. Hum Brain Mapp. 2008;29(1):82-96. doi:10.1002/hbm.20360 17318831 PMC6871049

[26] Du R, Zhou Q, Hu T, . A landmark-based approach to locate symptom-specific transcranial magnetic stimulation targets of depression. Front Psychol. 2022;13:919944. doi:10.3389/fpsyg.2022.919944 36118495 PMC9480500

[27] Lynch CJ, Elbau IG, Ng TH, . Automated optimization of TMS coil placement for personalized functional network engagement. Neuron. 2022;110(20):3263-3277.e4. doi:10.1016/j.neuron.2022.08.012 36113473 PMC11446252

[28] Numssen O, van der Burght CL, Hartwigsen G. Revisiting the focality of non-invasive brain stimulation—implications for studies of human cognition. Neurosci Biobehav Rev. 2023;149:105154. doi:10.1016/j.neubiorev.2023.105154 37011776 PMC10186117

[29] Opitz A, Legon W, Rowlands A, Bickel WK, Paulus W, Tyler WJ. Physiological observations validate finite element models for estimating subject-specific electric field distributions induced by transcranial magnetic stimulation of the human motor cortex. Neuroimage. 2013;81:253-264. doi:10.1016/j.neuroimage.2013.04.067 23644000

[30] Zhang BBB, Stöhrmann P, Godbersen GM, . Normal component of TMS-induced electric field is correlated with depressive symptom relief in treatment-resistant depression. Brain Stimul. 2022;15(5):1318-1320. doi:10.1016/j.brs.2022.09.006 36130678

[31] Qi S, Calhoun VD, Zhang D, . Links between electroconvulsive therapy responsive and cognitive impairment multimodal brain networks in late-life major depressive disorder. BMC Med. 2022;20(1):477. doi:10.1186/s12916-022-02678-6 36482369 PMC9733153

[32] Kim NY, Hsu J, Talmasov D, . Lesions causing hallucinations localize to one common brain network. Mol Psychiatry. 2021;26(4):1299-1309. doi:10.1038/s41380-019-0565-3 31659272

[33] Fox MD. Mapping symptoms to brain networks with the human connectome. N Engl J Med. 2018;379(23):2237-2245. doi:10.1056/NEJMra1706158 30575457

[34] Slotema CW, Blom JD, de Weijer AD, . Can low-frequency repetitive transcranial magnetic stimulation really relieve medication-resistant auditory verbal hallucinations? negative results from a large randomized controlled trial. Biol Psychiatry. 2011;69(5):450-456. doi:10.1016/j.biopsych.2010.09.051 21144499

[35] Vercammen A, Knegtering H, Liemburg EJ, den Boer JA, Aleman A. Functional connectivity of the temporo-parietal region in schizophrenia: effects of rTMS treatment of auditory hallucinations. J Psychiatr Res. 2010;44(11):725-731. doi:10.1016/j.jpsychires.2009.12.011 20189190

[36] Thielscher A, Antunes A, Saturnino GB. Field modeling for transcranial magnetic stimulation: a useful tool to understand the physiological effects of TMS? Annu Int Conf IEEE Eng Med Biol Soc. 2015:222-225. doi:10.1109/EMBC.2015.7318340 26736240

[37] Puonti O, Van Leemput K, Saturnino GB, Siebner HR, Madsen KH, Thielscher A. Accurate and robust whole-head segmentation from magnetic resonance images for individualized head modeling. Neuroimage. 2020;219:117044. doi:10.1016/j.neuroimage.2020.117044 32534963 PMC8048089

[38] Gomez LJ, Dannhauer M, Peterchev AV. Fast computational optimization of TMS coil placement for individualized electric field targeting. Neuroimage. 2021;228:117696. doi:10.1016/j.neuroimage.2020.117696 33385544 PMC7956218

[39] Vöröslakos M, Takeuchi Y, Brinyiczki K, . Direct effects of transcranial electric stimulation on brain circuits in rats and humans. Nat Commun. 2018;9(1):483. doi:10.1038/s41467-018-02928-3 29396478 PMC5797140

[40] Aleman A, Sommer IE, Kahn RS. Efficacy of slow repetitive transcranial magnetic stimulation in the treatment of resistant auditory hallucinations in schizophrenia: a meta-analysis. J Clin Psychiatry. 2007;68(3):416-421. doi:10.4088/JCP.v68n0310 17388712

[41] Kraemer HC, Kupfer DJ. Size of treatment effects and their importance to clinical research and practice. Biol Psychiatry. 2006;59(11):990-996. doi:10.1016/j.biopsych.2005.09.014 16368078

[42] Weigand A, Horn A, Caballero R, . Prospective validation that subgenual connectivity predicts antidepressant efficacy of transcranial magnetic stimulation sites. Biol Psychiatry. 2018;84(1):28-37. doi:10.1016/j.biopsych.2017.10.028 29274805 PMC6091227

[43] Zhang Y, Liang W, Yang S, Dai P, Shen L, Wang C. Repetitive transcranial magnetic stimulation for hallucination in schizophrenia spectrum disorders: a meta-analysis. Neural Regen Res. 2013;8(28):2666-2676. doi:10.3969/j.issn.1673-5374.2013.28.009 25206578 PMC4146020

[44] Lefaucheur JP, Aleman A, Baeken C, . Evidence-based guidelines on the therapeutic use of repetitive transcranial magnetic stimulation (rTMS): an update (2014-2018). Clin Neurophysiol. 2020;131(2):474-528. doi:10.1016/j.clinph.2019.11.002 31901449

[45] Montagne-Larmurier A, Etard O, Razafimandimby A, Morello R, Dollfus S. Two-day treatment of auditory hallucinations by high frequency rTMS guided by cerebral imaging: a 6 month follow-up pilot study. Schizophr Res. 2009;113(1):77-83. doi:10.1016/j.schres.2009.05.006 19505799

[46] de Weijer AD, Sommer IE, Lotte Meijering A, . High frequency rTMS; a more effective treatment for auditory verbal hallucinations? Psychiatry Res. 2014;224(3):204-210. doi:10.1016/j.pscychresns.2014.10.007 25453990

[47] Wang Y, Yang Y, Xu W, . Heterogeneous brain abnormalities in schizophrenia converge on a common network associated with symptom remission. Schizophr Bull. 2024;50(3):545-556. doi:10.1093/schbul/sbae003 38253437 PMC11059819

[48] Wang L, Chen X, Wu Y, . Intermittent theta burst stimulation (iTBS) adjustment effects of schizophrenia: results from an exploratory outcome of a randomized double-blind controlled study. Schizophr Res. 2020;216:550-553. doi:10.1016/j.schres.2019.12.008 31926810

[49] Brunelin J, Mondino M, Gassab L, . Examining transcranial direct-current stimulation (tDCS) as a treatment for hallucinations in schizophrenia. Am J Psychiatry. 2012;169(7):719-724. doi:10.1176/appi.ajp.2012.11071091 22581236

[50] Brady RO Jr, Gonsalvez I, Lee I, . Cerebellar-prefrontal network connectivity and negative symptoms in schizophrenia. Am J Psychiatry. 2019;176(7):512-520. doi:10.1176/appi.ajp.2018.18040429 30696271 PMC6760327

[51] Catani M, Craig MC, Forkel SJ, . Altered integrity of perisylvian language pathways in schizophrenia: relationship to auditory hallucinations. Biol Psychiatry. 2011;70(12):1143-1150. doi:10.1016/j.biopsych.2011.06.013 21798516

[52] Si T, Shi C, Sun L, Zhang Y, Zhang L. Assessment of the minimum clinically important difference in symptoms and functions of patients with acute schizophrenia: a post hoc analysis of an open-label, single-arm multicenter study. Front Psychiatry. 2021;12:653916. doi:10.3389/fpsyt.2021.653916 34012411 PMC8126618

[53] Sun J, Du R, Zhang B, . Minimal scanning duration for producing individualized repetitive transcranial magnetic stimulation targets. Brain Imaging Behav. 2022;16(6):2637-2646. doi:10.1007/s11682-022-00720-y 36181650

[54] Gratton C, Kraus BT, Greene DJ, . Defining individual-specific functional neuroanatomy for precision psychiatry. Biol Psychiatry. 2020;88(1):28-39. doi:10.1016/j.biopsych.2019.10.026 31916942 PMC7203002

[55] Dannhauer M, Gomez LJ, Robins PL, . Electric field modeling in personalizing transcranial magnetic stimulation interventions. Biol Psychiatry. 2024;95(6):494-501. doi:10.1016/j.biopsych.2023.11.022 38061463 PMC10922371

[56] Zanto TP, Jones KT, Ostrand AE, Hsu WY, Campusano R, Gazzaley A. Individual differences in neuroanatomy and neurophysiology predict effects of transcranial alternating current stimulation. Brain Stimul. 2021;14(5):1317-1329. doi:10.1016/j.brs.2021.08.017 34481095 PMC9492518

[57] Cotovio G, Oliveira-Maia AJ, Paul C, . Day-to-day variability in motor threshold during rTMS treatment for depression: clinical implications. Brain Stimul. 2021;14(5):1118-1125. doi:10.1016/j.brs.2021.07.013 34329797

