# Supplementary material for: Repetitive Transcranial Magnetic Stimulation for Auditory Verbal Hallucinations in Schizophrenia: A Randomized Clinical Trial
Source: JAMA Netw Open. 2024 Nov 11;7(11):e2444215. doi: 10.1001/jamanetworkopen.2024.44215 (PMC11555553; doi:10.1001/jamanetworkopen.2024.44215)
Supplement: Supplement 1. — Trial Protocol [file jamanetwopen-e2444215-s001.pdf]

Effect of Imaging-navigated Repetitive Transcranial Magnetic Stimulation for Auditory Verbal Hallucinations in Schizophrenia Patients: a randomized, double-blind, sham-controlled, clinical trial

1. Introduction

Auditory verbal hallucinations (AVH) is a common symptom of schizophrenia [1]. Approximately 70-80% of schizophrenia patients have AVH at initial presentation [2, 3], of which 25%-30% are irresponsive to antipsychotic medication and persist [4]. The persistence of AVH can significantly diminish the quality of life and elevate the risks of suicide and violence [5]. Consequently, alternative therapies are urgently needed to alleviate the severity and frequency of medication-resistant AVH. Repetitive transcranial magnetic stimulation (rTMS) as a non-invasive therapeutic strategy offers a potential treatment for AVH in schizophrenia patients. An increasing number of researchers place expectations on rTMS to ameliorate AVH, unfortunately, the efficacy of rTMS remains controversial [6-10]. The conflicting clinical responses may be attributed to the variability in rTMS sequences and target selections used across these studies.

The selection of the rTMS sequence is key to achieving more robust clinical efficacy. Although the efficacies in relieving AVH were mixed, a meta-analysis reported that 1-Hz rTMS on temporo-parietal junction (TPJ) cortex was statistically superior to sham treatment [11, 12]. The neuroimaging and neurophysiological studies have indicated a correlation between AVH and heightened cerebral activity in the left

temporal cortex, suggesting that rTMS efficacy in alleviating AVH may be explained by its potential inhibitory effects [13-15]. The continuous theta-burst stimulation (cTBS) paradigm has been proven to produce similar inhibitory effects on the motor system as 1-Hz rTMS [16]. However, the stimulation period of typical cTBS (~ 40s) is much shorter than 1-Hz rTMS (~ 10min) for the same number of pulses, which is an advantage for clinical application. The cTBS paradigm has been applied in the treatment of many neurological and psychiatric disorders. Furthermore, biochemical analysis [17] and neuroimaging studies [18] have reported that a three-block TBS protocol (inter-block interval = 15 min) may induce cumulative aftereffects. Thus, we hypothesized that this multi-cTBS protocol would be effective in alleviating AVH in schizophrenia patients.

Apart from the stimulation sequence, accurately identifying the stimulation target is crucial for enhancing clinical efficacy. Most studies investigating the potential of rTMS to alleviate AVH have chosen the TPJ cortex as a therapeutic target. Whereas, researchers typically rely on the T3P3 site of the international 10-20 system EEG electrode positioning to approximate the TPJ target. Considering the anatomical variability among patients, this approach proves imprecise in determining the exact TPJ target for each individual. In clinical rTMS studies, employing an image-guided navigation method can significantly enhance spatial precision, leading to more favorable outcomes [19, 20]. To address the clinical efficacy in treating AVH, we develop an optimized cTBS protocol that incorporates MRI navigation, an extended stimulation course (14 days), and a multi-block design (interval = 15 min). Our team

has discovered that the efficacy of optimized image-guided cTBS is significantly superior to both sham treatment and previous cTBS studies [10], as evidenced in an open-label trial [21]. To mitigate the placebo effect, we are assessing the efficacy of this optimized cTBS protocol through a randomized, double-blind, placebo-controlled trial.

2. Objective

Based on the background mentioned above, we will investigate the clinical efficacy of the modified cTBS protocol through a randomized double-blind placebo-controlled trial. We hypothesize that this modified cTBS protocol would reduce AVH symptoms superior to sham controls.

3. Study Design

We will conduct a single-site, randomized, double-blinded, placebo-controlled clinical trial with two arms. The eligible patients with schizophrenia will be recruited through posted flyers and physician referrals from Anhui Mental Health Center (Hefei, China). In the screening phase, inclusion and exclusion criteria are investigated. Patients who meet the inclusion criteria will provide their written informed consent to participate and be randomly assigned to one of the groups to receive either active or sham cTBS treatment. Involved patients, clinical raters, and all personnel responsible for the clinical care of patients remain blinded to the allocated condition. Only rTMS administrators have access to the randomization list, but they have minimal contact with the patients and no role in assessing AVH. After the inclusion and randomization processes, patients will undergo three daily sessions of cTBS treatment for 2 weeks,

with assessments scheduled at three different visits: baseline (week 0), post-treatment (week 2), and a follow-up visit (week 6). Baseline assessments are conducted the day before the first cTBS session. Post-treatment and follow-up assessments are conducted one day and four weeks after the last cTBS session, respectively. The Auditory Hallucination Rating Scale (AHRS), the Positive and Negative Syndrome Scale (PANSS), the Hamilton Anxiety Rating Scale with 14 items (HAMA), and the Hamilton Depression Rating Scale with 17 items (HAMD) are assessed by a trained rater at baseline, post-treatment), and follow-up visit. These scales are applied to assess the severity of AVH and other symptoms. Furthermore, demographic data, structure magnetic resonance imaging (sMRI) and functional magnetic resonance imaging (fMRI) data are collected at baseline. The sMRI data will be used to navigate stimulation in real-time. The specific trial procedures are presented in Figure 1.

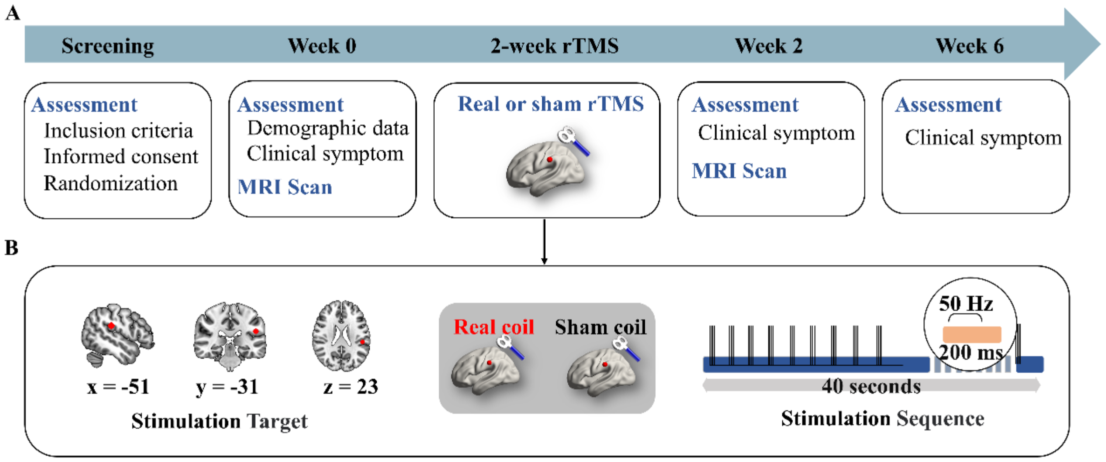

Figure 1. Schematic representation of the trial procedures. A, The trial consists of a 2-week rTMS treatment and three assessment visits at week 0, week 2, and week 6 (top). The clinical symptoms assessments include AHRS, PANSS, HAMA, and HAMD. B, The rTMS using continuous theta-burst stimulation (cTBS) paradigm will be performed with three daily sessions. The stimulation target on the

scalp will be navigated using Brainsight neuro-navigation software and individual structural MRI of each participant. The specific target of temporo-parietal junction (TPJ) cortex and the schematic diagram of the cTBS sequence as visualized at the bottom.

Abbreviations: MRI, magnetic resonance imaging; AHRS, Auditory Hallucination Rating Scale; PANSS, Positive and Negative Syndrome Scale; HAMA, Hamilton Anxiety Rating Scale; HAMD, Hamilton Depression Rating Scale; rTMS, repetitive transcranial magnetic stimulation.

4. Inclusion and Exclusion Criteria

Patients with the diagnosis of schizophrenia using the Structured Clinical Interview for the Diagnostic and Statistical Manual of Mental Disorders, the 4th Edition will be included, which will be applied by independent psychiatrists.

Here are some more detailed inclusion and exclusion criteria.

The inclusion criteria for patients included:

1) Aged 18 to 60 years old both genders;

2) patients with schizophrenia reported AVH at least 5 times per day using a written log or hand-held counter;

3) the AVH symptoms of patients have failed to achieve a clinical response to medication treatment (defined as an insufficient response to at least two antipsychotic agents, administered at adequate dosages for at least 6 weeks);

4) a stable dosage of antipsychotic medication for 2 weeks before inclusion and keep the dose stable for the duration of the study.

Exclusion criteria included:

1) Focal brain lesions on T1- or T2-weighted fluid-attenuated inversion-recovery MRI;

2) accompanied by other mental illness or history other than depression;

3) a history of severe head trauma or neurological disease;

4) recent aggression or other forms of behavioral dyscontrol;

5) a history of rTMS or electroconvulsive therapy;

6) with severe suicidal thoughts or behavior;

7) metal objects in the head or any other contraindication to MRI;

8) pregnant and current alcohol or drug abuse.

5. Randomization and Blinding

Patients will be randomly assigned to receive either active or sham cTBS treatments according to a computer-generated list by an unblinded investigator not involved with study ratings/analysis. The assignment will be executed by utilizing sealed opaque envelopes, each containing the code corresponding to the assigned group for every participant. To ensure the integrity of the double-blind procedure, patients, clinical raters, and all personnel responsible for the clinical care of patients will remain uninformed about the assigned condition until the end point of the study. Only rTMS administrators have access to the randomization list. For the sham cTBS group, we will use a sham coil with an appearance identical to the real coil, The only difference was that the sham coil generated only sound and sensations on the scalp similar to the real coil but no current, which prevents patients from identifying their

allocation to the rTMS group.

To measure blinding integrity, participants will be asked to guess which group they were randomly assigned to at the study endpoint. Participants are notified of the treatment condition at the endpoint. Participants in the placebo group will be given the opportunity to receive active treatment.

6. Interventions

Following randomization and baseline assessment, patients will undergo neuro-navigated cTBS treatment. The cTBS will be performed using a MagStim Rapid^2 transcranial magnetic stimulator (Magstim Company Ltd.) with a 70-mm air-cooled figure-of-eight coil. The frameless neuro-navigation system (Brainsight; Rogue Research, Montreal, QC, Canada) will be employed to position the coil for maximal field strength at the stimulation target regions for each participant.

According to previous studies, the left TPJ may be an effective target for AVH in schizophrenia patients [7, 8, 21]. Therefore, we restrict the stimulation target within the left TPJ, defined as a sphere of 6-mm radius centered at Montreal Neurological Institute (MNI) coordinates [−51, −31, 23] [22, 23]. This target is transformed into each participant's T1 space by applying an inverse matrix produced during T1 segmentation in SPM12 (www.fil.ion.ucl.ac.uk/spm) and an in-house TMStarget software [24]. Then, each individual's target is imported into the neuro-navigation system. The junction points of the figure-of-eight coil will be placed directly over the TPJ region. The entire stimulation process will be monitored by real-time MRI

navigation.

Before the first treatment, each participant’s resting motor threshold (RMT) will be determined according to a 5-step procedure over the primary motor cortex [25]. RMT was defined as the lowest intensity required to evoke a small response (>50 mV) in more than five of ten consecutive trials in the right first dorsal interosseous muscle. Participants will then undergo 3 daily sessions of cTBS treatment for 2 weeks. One session was 40 seconds in duration and consisted of triplet 50-Hz bursts, repeated at 5 Hz until a total of 600 pulses was reached [16]. To achieve cumulative aftereffects, this protocol was repeated 3 times (1,800 pulses in total) separated by two 15-minute breaks (controlled by a stopwatch) in line with previous methodological studies [17, 18]. During the 15-minute treatment interval, all patients were silent and rested with their eyes closed. The cTBS was delivered at 80% of the resting motor threshold (RMT) [26] or the highest intensity the stimulator could deliver for this protocol (50% of maximum output).

Sham cTBS treatments will be delivered with the same rTMS protocol using a sham coil (Magstim Company Ltd.), identical in appearance to the real one. The only difference is that the sham coil generates only sound and sensations on the scalp similar to the real coil but no current, which prevents patients from identifying group allocation.

Any adverse event related to the study (e.g., headache, pain, discomfort, or other harm caused by the intervention) will be recorded throughout the study. If serious adverse events occur, the participant will immediately be withdrawn from the study.

7. Measurement of Outcomes

7.1 Primary Outcome

The primary outcome is the changes in AHRS scores from baseline to post-treatment as well as follow-up.

7.2 Secondary Outcomes

Secondary outcomes measures will include: 1) the changes from baseline to post-treatment and follow-up in the PANSS, HAMA and HAMD score; 2) the number of responders at post-treatment and follow-up (responder is defined as ≥25% reduction from baseline in AHRS and PANSS total scores); 3) the adverse events and blinding integrity.

The flow chart for each patient is shown below (Table 1).

Table 1. Schedule of Events

Assessments	Screening and baseline (Week 0)	Post-treatment visit (Week 2)	Follow-up visit (Week 6)
**Inclusion/exclusion criteria**	×		
**Informed consent**	×		
**Randomization**	×		
**Demographic data**	×		
**MRI scan**	×	×	
**RMT**	×		
**AHRS**	×	×	×
**PANSS**	×	×	×

**HAMA**	×	×	×
**HAMD**	×	×	×
**Adverse events record**		×	×
**Blind integrity evaluate**			×

Abbreviations: MRI, magnetic resonance imaging; RMT, resting motor threshold; AHRS, Auditory Hallucination Rating Scale; PANSS, Positive and Negative Syndrome Scale; SAPS, Scale for Assessment of Positive Symptoms; SANS, the Scale for Assessment of Negative Symptoms; HAMA, Hamilton Anxiety Rating Scale; HAMD, Hamilton Depression Rating Scale.

8. Sample Size Calculation

The sample calculation is based on the AHRS, which is our study's primary outcome. According to a previous meta-analysis, which verified an efficacy favoring active low-frequency vs. sham rTMS with an effect size of 0.76 in the treatment-resistant AVH [12]. Setting a power of 80% and a 2-tailed α level of 5% for assessing AVH improvement, a sample size of 30 participants per group will be required. Additionally, a 10% attrition rate is considered, and the total sample size will be 66 participants.

9. MRI Parameters

The structural and resting-state functional MRI data for each patient at baseline. Structural and functional MRI data will be acquired utilizing a 3.0 T scanner (Discovery 750; GE Healthcare, Milwaukee, WI, USA) at the University of Science and Technology of China (Hefei, Anhui Province). Specifically, functional images (comprising 217 volumes) are acquired through a single-shot gradient-recalled echo

planar imaging sequence (repetition/echo time: 2400/30 ms; flip angle: 90º). Images of 46 transverse sections (field of view: 192 × 192 mm2; in-plane matrix: 64 × 64; section thickness: 3 mm without intersection gap) are acquired in parallel to the anteroposterior commissure line. Subsequently, high spatial resolution T1-weighted anatomic images will be obtained in the sagittal orientation employing a magnetization-prepared rapid gradient-echo sequence (repetition/echo time: 8.16/3.18 ms; flip angle: 12º; field of view: 256 × 256 mm2; 256 × 256 matrix; section thickness: 1 mm, without intersection gap; voxel size: 1 × 1 × 1 mm3; 188 sections). We will employ foam fillers and earplugs to minimize head motion and diminish scanner noise during the scanning phase. Participants are instructed to keep their eyes closed and maintain a state of rest without falling asleep while acquiring images.

10. Statistical Analysis

We will use the IBM SPSS Statistics version 23.0 for statistical analyses. For the primary outcome and all continuous secondary outcomes, we will use linear mixed-effects models to test the treatment efficacy. Time, treatment group, and time-by-treatment interaction will be included in the model as fixed effects. The individual participant intercept will be included in the model as random effects. For the binary outcomes of treatment response, we will use the Chi-squared test or Fisher exact test to compare between groups. P values are 2-sided, and $P < .05$ will be considered statistically significant. Effect sizes will be calculated as Cohen's d and odds ratios for continuous and binary outcomes, respectively. We also provided the number needed to

treat (NNT), to assess the effectiveness of a clinical intervention [27]. The missing data will be addressed using the multiple imputations method.

Reference:

1. Sommer, IE, Slotema, CW, Daskalakis, ZJ, et al. The treatment of hallucinations in schizophrenia spectrum disorders. Schizophr Bull 2012;38(4):704-714.
2. Andreasen, NC, Flaum, M. Schizophrenia: the characteristic symptoms. Schizophr Bull 1991;17(1):27-49.
3. Goghari, VM, Harrow, M, Grossman, LS, et al. A 20-year multi-follow-up of hallucinations in schizophrenia, other psychotic, and mood disorders. Psychol Med 2013;43(6):1151-1160.
4. Shergill, SS, Murray, RM, McGuire, PK. Auditory hallucinations: a review of psychological treatments. Schizophr Res 1998;32(3):137-150.
5. Hor, K, Taylor, M. Suicide and schizophrenia: a systematic review of rates and risk factors. J Psychopharmacol 2010;24(4 Suppl):81-90.
6. Hoffman, RE, Boutros, NN, Berman, RM, et al. Transcranial magnetic stimulation of left temporoparietal cortex in three patients reporting hallucinated "voices". Biol Psychiatry 1999;46(1):130-132.
7. Hoffman, RE, Gueorguieva, R, Hawkins, KA, et al. Temporoparietal transcranial magnetic stimulation for auditory hallucinations: safety, efficacy and moderators in a fifty patient sample. Biol Psychiatry 2005;58(2):97-104.
8. Bagati, D, Nizamie, SH, Prakash, R. Effect of augmentatory repetitive transcranial magnetic stimulation on auditory hallucinations in schizophrenia: randomized controlled study. Aust N Z J Psychiatry 2009;43(4):386-392.

9. Slotema, CW, Blom, JD, de Weijer, AD, et al. Can low-frequency repetitive transcranial magnetic stimulation really relieve medication-resistant auditory verbal hallucinations? Negative results from a large randomized controlled trial. Biol Psychiatry 2011;69(5):450-456.

10. Koops, S, van Dellen, E, Schutte, MJ, et al. Theta Burst Transcranial Magnetic Stimulation for Auditory Verbal Hallucinations: Negative Findings From a Double-Blind-Randomized Trial. Schizophr Bull 2016;42(1):250-257.

11. Slotema, CW, Blom, JD, van Lutterveld, R, et al. Review of the efficacy of transcranial magnetic stimulation for auditory verbal hallucinations. Biol Psychiatry 2014;76(2):101-110.

12. Aleman, A, Sommer, IE, Kahn, RS. Efficacy of slow repetitive transcranial magnetic stimulation in the treatment of resistant auditory hallucinations in schizophrenia: a meta-analysis. J Clin Psychiatry 2007;68(3):416-421.

13. Dierks, T, Linden, DE, Jandl, M, et al. Activation of Heschl's gyrus during auditory hallucinations. Neuron 1999;22(3):615-621.

14. Poulet, E, Brunelin, J, Bediou, B, et al. Slow transcranial magnetic stimulation can rapidly reduce resistant auditory hallucinations in schizophrenia. Biol Psychiatry 2005;57(2):188-191.

15. Reulbach, U, Bleich, S, Maihofner, C, et al. Specific and unspecific auditory hallucinations in patients with schizophrenia: a magnetoencephalographic study. Neuropsychobiology 2007;55(2):89-95.

16. Huang, YZ, Edwards, MJ, Rounis, E, et al. Theta burst stimulation of the human

motor cortex. Neuron 2005;45(2):201-206.

17. Volz, LJ, Benali, A, Mix, A, et al. Dose-dependence of changes in cortical protein expression induced with repeated transcranial magnetic theta-burst stimulation in the rat. Brain Stimul 2013;6(4):598-606.

18. Nettekoven, C, Volz, LJ, Kutscha, M, et al. Dose-dependent effects of theta burst rTMS on cortical excitability and resting-state connectivity of the human motor system. J Neurosci 2014;34(20):6849-6859.

19. Sack, AT, Cohen Kadosh, R, Schuhmann, T, et al. Optimizing functional accuracy of TMS in cognitive studies: a comparison of methods. J Cogn Neurosci 2009;21(2):207-221.

20. Sparing, R, Buelte, D, Meister, IG, et al. Transcranial magnetic stimulation and the challenge of coil placement: a comparison of conventional and stereotaxic neuronavigational strategies. Hum Brain Mapp 2008;29(1):82-96.

21. Chen, X, Ji, GJ, Zhu, C, et al. Neural Correlates of Auditory Verbal Hallucinations in Schizophrenia and the Therapeutic Response to Theta-Burst Transcranial Magnetic Stimulation. Schizophr Bull 2019;45(2):474-483.

22. Vercammen, A, Knegtering, H, den Boer, JA, et al. Auditory hallucinations in schizophrenia are associated with reduced functional connectivity of the temporo-parietal area. Biol Psychiatry 2010;67(10):912-918.

23. Vercammen, A, Knegtering, H, Liemburg, EJ, et al. Functional connectivity of the temporo-parietal region in schizophrenia: effects of rTMS treatment of auditory hallucinations. J Psychiatr Res 2010;44(11):725-731.

24. Ji, GJ, Yu, F, Liao, W, et al. Dynamic aftereffects in supplementary motor network following inhibitory transcranial magnetic stimulation protocols. Neuroimage 2017;149:285-294.

25. Schutter, DJ, van Honk, J. A standardized motor threshold estimation procedure for transcranial magnetic stimulation research. J ect 2006;22(3):176-178.

26. Plewnia, C, Zwissler, B, Wasserka, B, et al. Treatment of auditory hallucinations with bilateral theta burst stimulation: a randomized controlled pilot trial. Brain Stimul 2014;7(2):340-341.

27. Kraemer, HC, Kupfer, DJ. Size of treatment effects and their importance to clinical research and practice. Biol Psychiatry 2006;59(11):990-996.
