# Supplementary material for: Repetitive Transcranial Magnetic Stimulation for Auditory Verbal Hallucinations in Schizophrenia: A Randomized Clinical Trial
Source: JAMA Netw Open. 2024 Nov 11;7(11):e2444215. doi: 10.1001/jamanetworkopen.2024.44215 (PMC11555553; doi:10.1001/jamanetworkopen.2024.44215)
Supplement: Supplement 2. — eMethods 1. MRI Data Acquisition eMethods 2. Treatment Protocol eMethods 3. Electric Field Modeling Method eMethods 4. Missing Data and Sensitivity Analysis eResults 1. Secondary Outcomes of rTMS Treatment eResults 2. Sensitivity Analysis of rTMS Treatment Outcomes eResults 3. Estimating Clinical Efficacy in Sham Group eResults 4. Estimating Clinical Efficacy Using Different Confounders eTable 1. Outcomes of Sensitivity Analysis eTable 2. Regression Coefficients of Hierarchical Multiple Regression (6 Confounders) eTable 3. Correlations Between AHRS Reductions and Variables eTable 4. Regression Coefficients of Hierarchical Multiple Regression (1 Confounder) eTable 5. Adverse Events eFigure 1. Individualized AVH Network Mapping Method eFigure 2. Clinical Secondary Outcomes of rTMS Treatment eFigure 3. Estimating Clinical Efficacy in Sham Group eReferences [file jamanetwopen-e2444215-s002.pdf]

Supplementary Online Content

Hua Q, Wang L, He K, et al. Repetitive transcranial magnetic stimulation for auditory verbal hallucinations in schizophrenia: a randomized clinical trial. *JAMA Netw Open*. 2023;6(11):e2444215s. doi:10.1001/jamanetworkopen.2024.44215

eMethods 1. MRI Data Acquisition
eMethods 2. Treatment Protocol
eMethods 3. Electric Field Modeling Method
eMethods 4. Missing Data and Sensitivity Analysis
eResults 1. Secondary Outcomes of rTMS Treatment
eResults 2. Sensitivity Analysis of rTMS Treatment Outcomes
eResults 3. Estimating Clinical Efficacy in Sham Treatment Group
eResults 4. Estimating Clinical Efficacy Using Different Confounders
eTable 1. Outcomes of Sensitivity Analysis
eTable 2. Regression Coefficients of Hierarchical Multiple Regression (6 Confounders)
eTable 3. Correlations Between AHRS Reductions and Variables
eTable 4. Regression Coefficients of Hierarchical Multiple Regression (1 Confounder)
eTable 5. Adverse Events
eFigure 1. Individualized AVH Network Mapping Method
eFigure 2. Clinical Secondary Outcomes of rTMS Treatment
eFigure 3. Estimating Clinical Efficacy in Sham Treatment Group
eReferences

This supplementary material has been provided by the authors to give readers additional information about their work.

©2024 Hua Q et al. *JAMA Network Open*.

eMethods 1. MRI Data Acquisition

eMethods 2. Treatment Protocol

One session of continuous theta burst stimulation (cTBS) treatment was 40 seconds in duration and consisted of triplet 50-Hz bursts, repeated at 5 Hz until 600 pulses was reached [1]. To achieve cumulative aftereffects, this protocol was repeated three times (1,800 pulses in total) separated by two 15-minute breaks (controlled by a stopwatch) consistent with previous methodological studies [2, 3]. The cTBS was delivered at 80% of the resting motor threshold (RMT) [4] or the highest intensity the stimulator could deliver for this protocol (50% of maximum output). The RMT was determined at each visit according to a five-step procedure [5].

The stimulation target in the left temporoparietal junction (TPJ) was defined as a sphere of 6-mm radius centered at the Montreal Neurological Institute (MNI) coordinates [−51, −31, 23] [6-8]. This target was transformed into each participant's T1 space by applying an inverse matrix produced during T1 segmentation in SPM12 (https://www.fil.ion.ucl.ac.uk/spm/) using an in-house TMStarget software [9]. Then, each individual's target was imported into a frameless neuro-navigation system (Brainsight; Rogue Research, Montreal, Quebec, Canada). The coil was real-time guided tangentially to the skull, pointing forward, with the center over the target sphere.

eMethods 3. Electric Field Modeling Method

We computed the seed-to-whole brain functional connectivity using the resting-state

functional MRI data from 652 healthy subjects. MRI data from 652 healthy subjects (336 women [52%]; mean [SD] age, 22.9 [5.55]) were previously collected for 'local to network mapping' [10, 11]. The MRI scanning parameters for these 652 healthy subjects were the same as those used for patients with schizophrenia in the current study (see eMethod 1 in Supplement 2).

eMethods 4. Missing Data and Sensitivity Analysis

Patients who dropped out after randomization and at least one session of treatment were also analyzed. Linear mixed-effects models were used to test treatment efficacies, with no imputation of missing data in the main analysis. To ensure the robustness of our findings, a sensitivity analysis was conducted using the multiple imputation method to address missing data.

eResults 1. Secondary Outcomes of rTMS Treatment

The response to rTMS treatment was also defined as a ≥ 25% reduction from baseline in the PANSS total score. We found that the active rTMS group had higher response rates than the sham treatment group at week 2 (11 of 32 [34%] vs. 3 of 30 [10%]; $\chi^2 = 5.26$, $P = .02$; OR = 4.71, 95% CI, 1.11–16.98; NNT = 4.10, 95% CI, 2.24–94.27; eFigure 2B in Supplement 2) and week 6 (9 of 28 [32%] vs. 0 of 18 [0%]; $\chi^2 = 3.61$, $P = .007$; NNT = 3.0, 95% CI,1.85–16.20; eFigure 2B in Supplement 2).

When defining response to rTMS treatment using various cut-off points (e.g. 10%, 20%, 30%, and increasing incrementally up to 100%) for reductions in AHRS and

©2024 Hua Q et al. *JAMA Network Open*.

PANSS total scores, we observed higher response rates in the active group than the sham treatment group (eFigure 2C, D in Supplement 2).

eResults 2. Sensitivity Analysis of rTMS Treatment Outcomes

Sixty-two patients were included in the intent-to-treat (ITT) analysis. Data from these patients were complete at baseline and week 2 assessments. At week 6, AHRS and PANSS data were missing for 16 patients (26%), while HAMA and HAMD data were missing for 19 patients (31%). We addressed these missing data using the multiple imputation method with five imputations.

Sensitivity analysis revealed a significant time-by-group interaction ($F_{1,48.62} = 43.34$, $P < .001$) for AHRS at week 6. Post hoc tests revealed that patients in the active group showed a greater reduction in AHRS scores compared to the sham treatment group (group difference, 8.05; 95% CI, 5.65–10.45; $P < .001$; Cohen's $d = 1.67$; 95% CI, 1.09–2.25; eTable 1 in Supplement 2). Additionally, the sensitivity analysis showed higher response rates in the active group than the sham treatment group at week 6 (16 of 32 [50%] vs. 1 of 30 [3%]; $\chi^2 = 16.94$, P < .001; OR = 29.0, 95% CI, 3.51–239.31; NNT = 2.14, 95% CI,1.55–4.36; eTable 1 in Supplement 2).

Sensitivity analyses also showed significant time-by-group interactions for the PANSS total score, PANSS positive score, PANSS negative score, PANSS general score, HAMA, and HAMD score at week 6. Post hoc tests revealed that patients receiving active rTMS showed greater reductions in these scale scores compared to the sham treatment group (eTable 1 in Supplement 2). The results of sensitivity analysis

are consistent with our main analysis across all scales (eTable 1 in Supplement 2).

eResults 3. Estimating Clinical Efficacy in Sham Treatment Group

The e-field strength within the AVH network (r = 0.09, *P* = .64; eFigure 3) or e-field strength within the TPJ target region (r = −0.07, *P* = .74) were not correlated with the reduction in AHRS scores in the sham treatment group. Therefore, we did not conduct hierarchical multiple regression analysis.

eResults 4. Estimating Clinical Efficacy Using Different Confounders

To assess whether the TMS-induced e-field strength was associated with clinical efficacy, hierarchical multiple regression analysis was conducted [12]. In the main text, confounders in model 1 were selected based on prior research and theoretical foundations. To test the robustness of the estimation findings, we reconducted hierarchical multiple regression analysis including confounders based on Pearson's correlation analysis. Initially, Pearson's correlation analyses were performed to identify possible relationships between the dependent variable and confounders. The reduction in AHRS scores was the dependent variable of the model. Confounders included baseline data (i.e., sex, age, years of education, duration of illness, olanzapine equivalent, and baseline AHRS score). Model 1 included confounders that significantly correlated with reductions in AHRS scores. Model 2 included both confounders and estimation factors. The estimation factors was the TMS-induced e-field strength within the TPJ target region or AVH network. The estimation ability of the two models was

compared by an *F*-test. Then, a *t*-test was used to assess the contribution of the factors in estimating the dependent variable.

The reductions in AHRS scores were correlated with sex (r = 0.54, P = .001). Age, education years, illness duration, olanzapine equivalent, baseline AHRS score, and e-field strength within the TPJ target were not significantly correlated with reductions in AHRS scores (see eTable 3 in Supplement 2). Therefore, model 1 included sex (R^2 = 0.30, F = 12.63, P =.001). The reduction in AHRS scores was significantly correlated with e-field strength within the AVH network (r = 0.54, P =.001), but not with the e-field strength within the target region (r = 0.14, P =.44). Therefore, the e-field strength within the AVH network was added to model 2 as a variable of estimation, while e-field strength within the target region was not added. Model 2 included sex and e-field strength within the AVH network (R^2 = 0.47, F = 13.05, $P < .001$). The e-field strength within the AVH network significantly enhanced the ability of the model to estimate outcomes (ΔR^2 = 0.18, F = 9.78, P =.004; B = 2.58, t = 3.13, P = .004). Detailed regression coefficients of hierarchical multiple regression are given in eTable 4 in Supplement 2.

©2024 Hua Q et al. *JAMA Network Open*.

eTable 1. Outcomes of Sensitivity Analysis

Outcome	Patient group, mean (SD)		Sham treatment vs active rTMS group			
	**Sham treatment (n=30)**	**Active rTMS (n=32)**	**Difference (95% CI)[a]**	**Time-by-group[b]**	***p* value**	**Effect size, Cohen *d* (95% CI)**
**Secondary Outcome at week 6**						
Change in AHRS[c]	-0.48 (4.82)	-8.53 (4.81)	8.05 (5.65-10.45)	$F_{1,60}$=43.34	<.001	1.67 (1.09-2.25)
Change in PANSS total[d]	2.42 (10.08)	-13.04 (10.07)	15.46 (10.44-20.48)	$F_{1,60}$=36.30	<.001	1.53 (0.97-2.10)
Change in PANSS positive[e]	0.48 (4.11)	-4.25 (4.13)	4.73 (2.68-6.78)	$F_{1,60}$=24.87	<.001	1.15 (0.61-1.69)
Change in PANSS negative[e]	0.74 (3.67)	-4.15 (3.68)	4.89 (3.06-6.72)	$F_{1,60}$=14.41	<.001	1.33 (0.78-1.88)
Change in PANSS general[f]	1.28 (5.09)	-4.65 (5.09)	5.93 (3.39-8.47)	$F_{1,60}$=5.05	.03	1.16 (0.63-1.70)
Change in HAMA[g]	0.90 (4.16)	-2.40 (4.13)	3.30 (1.23-5.37)	$F_{1,60}$=9.80	.003	0.80 (0.28-1.31)
Change in HAMD[h]	0.64 (2.85)	-1.70 (2.83)	2.34 (0.93-3.75)	$F_{1,60}$=10.33	.002	0.82 (0.31-1.34)
≥25% Improvement[i]	1 (3)	16 (50)	2.14 (1.55-4.36)	χ^2=16.94	.001	29.0 (3.51-239.31)

Abbreviations: AHRS, Auditory Hallucination Rating Scale; HAMA, Hamilton Anxiety Rating Scale; HAMD, Hamilton Depression Rating Scale; OR, odds ratio; PANSS, Positive and Negative Syndrome Scale; rTMS, repetitive transcranial magnetic stimulation.

[a] Changes in scale score were the estimated marginal mean from linear mixed-effects models. The differences were calculated based on the estimated marginal mean.

[b] Time-by-group interaction of the linear mixed-effects model.

[c] Scores range, 0 to 41; higher scores indicate more severe symptoms.

[d] Scores range, 30 to 210; higher scores indicate more severe symptoms.

[e] cores range, 7 to 49; higher scores indicate more severe symptoms.

[f] Scores range, 16 to 112; higher scores indicate more severe symptoms.

[g] Scores range, 0 to 56; higher scores indicate more severe symptoms.

[h] Scores range, 0 to 50; higher scores indicate more severe symptoms.

©2024 Hua Q et al. *JAMA Network Open*.

[i] Shows No. (%) for patient group, number needed to treat rather than difference, and OR (95% CI) rather than Cohen *d*.

©2024 Hua Q et al. *JAMA Network Open*.

eTable 2. Regression Coefficients of Hierarchical Multiple Regression (6 Confounders)

	Model 1			Model 2		
**Variables**	**B**	**Beta**	**t/P**	**B**	**Beta**	**t/*P***
Sex	25.90	0.50	2.86/.008	21.01	0.41	2.77/.01
Age	0.28	0.10	0.31/.76	1.08	0.38	1.36/.19
Education years	1.28	0.17	0.91/.37	2.10	0.27	1.76/.09
Illness duration	0.17	0.03	0.10/.93	-1.33	-0.24	-0.86/.40
Olanzapine equivalent	0.09	0.05	0.31/.76	0.17	0.11	0.75/.46
Baseline AHRS score	-0.32	-0.08	-0.47/.64	-0.29	-0.07	-0.51/.62
E-field in AVH network				3.12	0.53	3.58/.002

Abbreviations: E-field, electric field; AVH, Auditory verbal hallucinations; AHRS, Auditory Hallucination Rating Scale.

B (unstandardized coefficient) represents the change in the dependent variable (reduction in AHRS scores) for a one-unit change in the estimation factors, holding other variables constant. Beta (standardized coefficient) refers to how many standard deviations a dependent variable will change, per standard deviation increase in the estimation factors. Beta is unitless and allows for comparison between variables on different scales.

©2024 Hua Q et al. *JAMA Network Open*.

eTable 3. Correlations Between AHRS Reductions and Variables

Variables	Active rTMS		Sham treatment	
	**r**	***P*[a]**	**r**	***P*[a]**
Sex	0.54	.001	0.18	.35
Age	0.13	.47	-0.32	.09
Education years	0.13	.47	0.09	.64
Illness duration	0.20	.28	-0.14	.46
Olanzapine equivalent	0.07	.72	-0.06	.74
Baseline AHRS score	-0.20	.26	-0.33	.08
E-field of TPJ target	0.14	.44	-0.07	.74
E-field of AVH network	0.54	.001	0.09	.64

Abbreviations: AHRS, Auditory Hallucination Rating Scale; E-field, electric field; TPJ, temporoparietal junction; AVH, Auditory verbal hallucinations.

[a] Pearson correlation analysis.

©2024 Hua Q et al. *JAMA Network Open*.

eTable 4. Regression Coefficients of Hierarchical Multiple Regression (1 Confounder)

	Model 1			Model 2		
**Variables**	**B**	**Beta**	**t/P**	**B**	**Beta**	**t/*P***
Sex	28.03	0.54	3.55/.001	22.69	0.44	3.18/.004
E-field in AVH network				2.58	0.43	3.13/.004

Abbreviations: E-field, electric field; AVH, Auditory verbal hallucinations; AHRS, Auditory Hallucination Rating Scale.

B (unstandardized coefficient) represents the change in the dependent variable (reduction in AHRS scores) for a one-unit change in the estimation factors, holding other variables constant. Beta (standardized coefficient) refers to how many standard deviations a dependent variable will change, per standard deviation increase in the estimation factors. Beta is unitless and allows for comparison between variables on different scales.

©2024 Hua Q et al. *JAMA Network Open*.

eTable 5. Adverse Events

Adverse Event	Frequency of Adverse Effects, No. (%)		*P* value
	**Sham treatment (n=30)**	**Active rTMS (n=32)**	
Headache	3 (10)	5 (16)	.78[a]
Scalp pain	5 (17)	8 (25)	.42[a]
Burning sensation	4 (13)	8 (25)	.25[a]
Back/neck pain	3 (10)	6 (19)	.54[a]
Tinnitus	0 (0)	2 (6)	.49[b]
Sleepiness	10 (33)	9 (28)	.66[a]
Epileptic seizures	0 (0)	0 (0)	>.99[b]
Unrelated accidents	0 (0)	0 (0)	>.99[b]

[a] Chi-square tests were used to compare differences in the adverse events between groups.

[b] If the expected frequencies in any cell of the contingency table were less than 1, Fisher exact tests were used to compare differences in the adverse events between groups.

©2024 Hua Q et al. *JAMA Network Open*.

eFigure 1. Individualized AVH Network Mapping Method

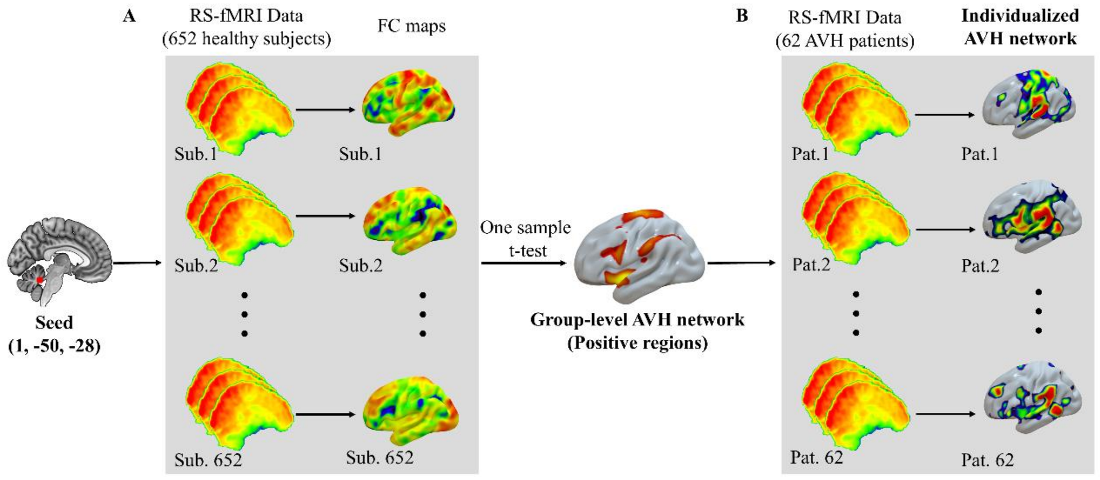

A, The hub region of the AVH network, identified by a prior lesion network mapping study [13], was used as a seed (MNI coordinate, 1, -50, -28). We computed the seed-to-whole brain FC on the rs-fMRI data of 652 healthy subjects, generating 652 FC maps. A one-sample *t*-test on 652 FC maps obtained the connectivity pattern of the hub region, termed the 'group-level network'. B, We used the positive part of the group-level AVH network as a seed map and computed its whole brain functional connectivity on each patient's rs-fMRI data. Voxels positively correlated with the seed map (uncorrected $P < .05$) constituted each patient's individualized AVH network. RS-fMRI indicates resting-state functional MRI. FC indicates functional connectivity. AVH indicates auditory verbal hallucination. Sub indicates subject. Pat indicates patient.

©2024 Hua Q et al. *JAMA Network Open*.

eFigure 2. Clinical Secondary Outcomes of rTMS Treatment

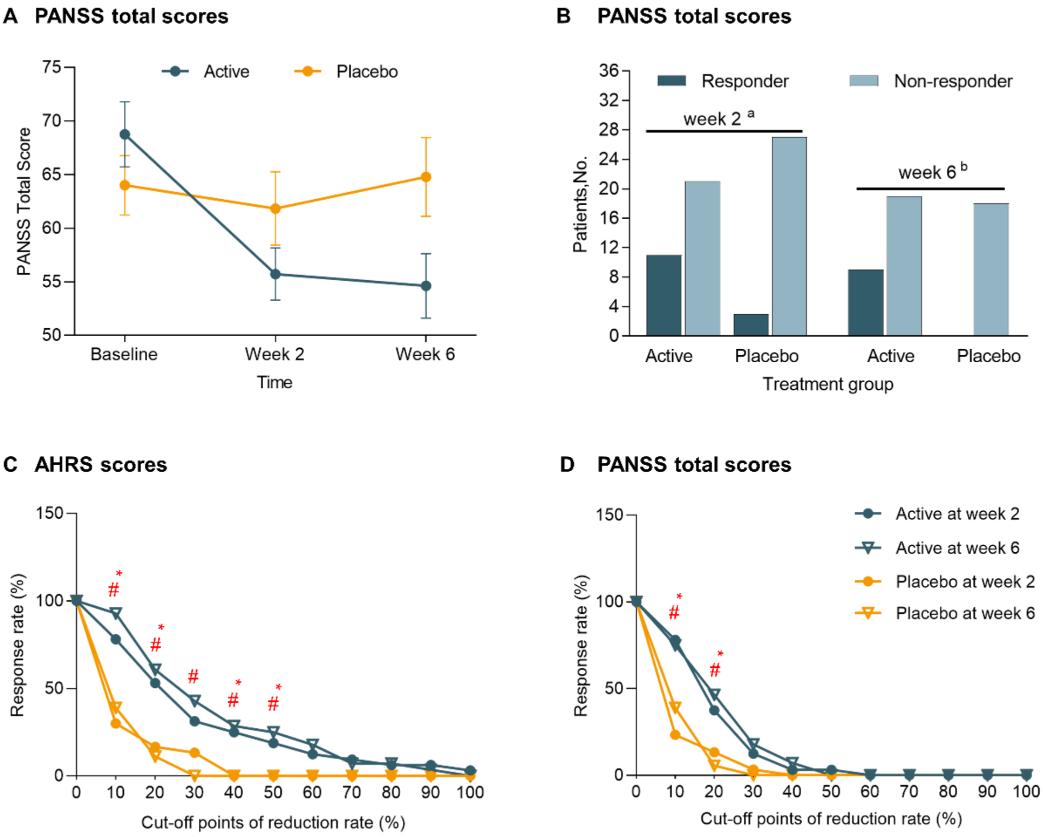

A, Changes in PANSS total scores over time in the active and sham treatment groups. Greater reductions were found in the active group than in the sham treatment group at both week 2 and week 6. The error bars indicate 1 SEM. B, The response rate for PANSS total scores was significantly higher in the active group than the sham treatment group at week 2 and week 6. C, The response rates for AHRS in the active group were higher than that of the sham treatment group under various cut-off points. D, The response rate for PANSS total scores in the active group was higher than that of the sham treatment group under various cut-off points. [*] and [#] indicate that the differences in response rates between the active and sham treatment groups were significant at week 2 and week 6, respectively. rTMS indicates repetitive transcranial magnetic stimulation. PANSS indicates the Positive and Negative Syndrome Scale. AHRS indicates the Auditory Hallucination Rating Scale. [a] $P < .05$; [b] $P < .01$.

©2024 Hua Q et al. *JAMA Network Open*.

eFigure 3. Estimating Clinical Efficacy in Sham Treatment Group

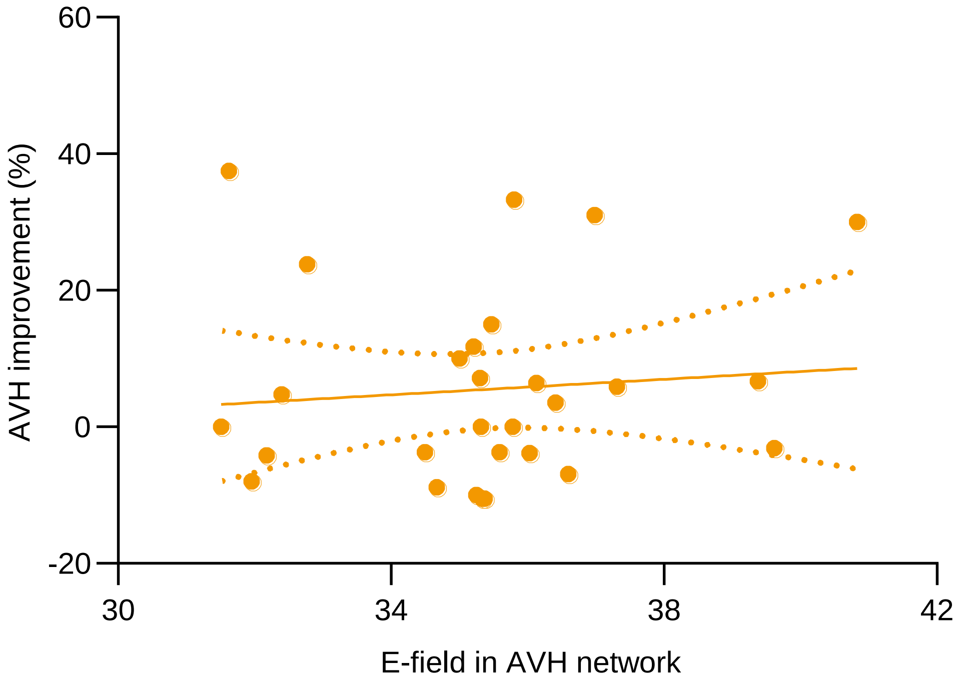

The reduction in AHRS score was not correlated with e-field strength within each patient's individualized AVH network (r = −0.09, P = .64) in the sham treatment group. AHRS indicates the Auditory Hallucination Rating Scale. E-field the indicates electric field. AVH indicates auditory verbal hallucinations.

©2024 Hua Q et al. *JAMA Network Open*.

eReferences.

1. Huang, YZ, Edwards, MJ, Rounis, E, et al. Theta burst stimulation of the human motor cortex. Neuron 2005;45(2):201-206.
2. Nettekoven, C, Volz, LJ, Kutscha, M, et al. Dose-dependent effects of theta burst rTMS on cortical excitability and resting-state connectivity of the human motor system. The Journal of neuroscience : the official journal of the Society for Neuroscience 2014;34(20):6849-6859.
3. Volz, LJ, Benali, A, Mix, A, et al. Dose-dependence of changes in cortical protein expression induced with repeated transcranial magnetic theta-burst stimulation in the rat. Brain Stimul 2013;6(4):598-606.
4. Plewnia, C, Zwissler, B, Wasserka, B, et al. Treatment of auditory hallucinations with bilateral theta burst stimulation: a randomized controlled pilot trial. Brain stimulation 2014;7(2):340-341.
5. Schutter, DJ, van Honk, J. A standardized motor threshold estimation procedure for transcranial magnetic stimulation research. The journal of ECT 2006;22(3):176-178.
6. Vercammen, A, Knegtering, H, den Boer, JA, et al. Auditory hallucinations in schizophrenia are associated with reduced functional connectivity of the temporo-parietal area. Biological psychiatry 2010;67(10):912-918.
7. Vercammen, A, Knegtering, H, Liemburg, EJ, et al. Functional connectivity of the temporo-parietal region in schizophrenia: effects of rTMS treatment of auditory hallucinations. Journal of psychiatric research 2010;44(11):725-731.
8. Chen, X, Ji, GJ, Zhu, C, et al. Neural correlates of auditory verbal hallucinations in schizophrenia and the therapeutic response to theta-burst transcranial magnetic stimulation.

©2024 Hua Q et al. *JAMA Network Open*.

Schizophrenia bulletin 2019;45(2):474-483.

9. Ji, GJ, Yu, F, Liao, W, et al. Dynamic aftereffects in supplementary motor network following inhibitory transcranial magnetic stimulation protocols. Neuroimage 2017;149:285-294.

10. Ji, GJ, Zalesky, A, Wang, Y, et al. Linking Personalized Brain Atrophy to Schizophrenia Network and Treatment Response. Schizophr Bull 2023;49(1):43-52.

11. Yang, Y, Xu, W, Wang, Y, et al. Heterogeneous Brain Atrophy Sites in Anxiety Disorders Map to a Common Brain Network. Depression and Anxiety 2024;2024(1):3827870.

12. Weigand, A, Horn, A, Caballero, R, et al. Prospective Validation That Subgenual Connectivity Predicts Antidepressant Efficacy of Transcranial Magnetic Stimulation Sites. Biol Psychiatry 2018;84(1):28-37.

13. Kim, NY, Hsu, J, Talmasov, D, et al. Lesions causing hallucinations localize to one common brain network. Mol Psychiatry 2021;26(4):1299-1309.

©2024 Hua Q et al. *JAMA Network Open*.
