# Supplementary material for: Repetitive Transcranial Magnetic Stimulation for Auditory Verbal Hallucinations in Schizophrenia: A Randomized Clinical Trial
Source: JAMA Netw Open. 2024 Nov 11;7(11):e2444215. doi: 10.1001/jamanetworkopen.2024.44215 (PMC11555553; doi:10.1001/jamanetworkopen.2024.44215)
Supplement: Supplement 3. — Data Sharing Statement [file jamanetwopen-e2444215-s003.pdf]

Data Sharing Statement

Hua. Repetitive Transcranial Magnetic Stimulation for Auditory Verbal Hallucinations in Schizophrenia. *JAMA Netw Open*. Published November 11, 2024.
doi:10.1001/jamanetworkopen.2024.44215

Data
Additional Information: Name of the trial registry: ClinicalTrials.gov; Registry's URL: https://clinicaltrials.gov/; Registration number: NCT02863094
Data available: Yes
Data types: Participant data with identifiers
How to access data: The data that support the findings of this study are available from the corresponding author, Gong-Jun Ji (jigongjun@163.com), upon reasonable request.
When available: With publication

Supporting Documents
Document types: None

Additional Information
Who can access the data: Researchers who provide a methodologically sound proposal that includes a protocol and a statistical analysis plan, and is not in conflict with the investigators' publication plan.
Types of analyses: For a specified purpose.
Mechanisms of data availability: To gain access, data requestors will need to sign a data access agreement.
